# Crosstalk of Magnesium and Serum Lipids in Dyslipidemia and Associated Disorders: A Systematic Review

**DOI:** 10.3390/nu13051411

**Published:** 2021-04-22

**Authors:** Mihnea-Alexandru Găman, Elena-Codruța Dobrică, Matei-Alexandru Cozma, Ninel-Iacobus Antonie, Ana Maria Alexandra Stănescu, Amelia Maria Găman, Camelia Cristina Diaconu

**Affiliations:** 1 Faculty of Medicine, “Carol Davila” University of Medicine and Pharmacy, 050474 Bucharest, Romania; alexandrazotta@yahoo.com; 2 Department of Hematology, Center of Hematology and Bone Marrow Transplantation, Fundeni Clinical Institute, 022328 Bucharest, Romania; 3 Department of Pathophysiology, University of Medicine and Pharmacy of Craiova, 200349 Craiova, Romania; codrutadobrica@yahoo.com; 4 Department of Dermatology, “Elias” University Emergency Hospital, 011461 Bucharest, Romania; 5 Department of Gastroenterology, Colentina Clinical Hospital, 20125 Bucharest, Romania; matei.cozma@gmail.com; 6 Department of Internal Medicine, Clinical Emergency Hospital of Bucharest, 14461 Bucharest, Romania; antonieninel@yahoo.com; 7 Clinic of Hematology, Filantropia City Hospital, 200143 Craiova, Romania

**Keywords:** magnesium, magnesemia, hypomagnesemia, lipids, cholesterol, triglycerides, dyslipidemia, hyperlipidemia, diabetes, metabolic syndrome

## Abstract

Dyslipidemia is a significant threat to public health worldwide and the identification of its pathogenic mechanisms, as well as novel lipid-lowering agents, are warranted. Magnesium (Mg) is a key element to human health and its deficiency has been linked to the development of lipid abnormalities and related disorders, such as the metabolic syndrome, type 2 diabetes mellitus, or cardiovascular disease. In this review, we explored the associations of Mg (dietary intake, Mg concentrations in the body) and the lipid profile, as well as the impact of Mg supplementation on serum lipids. A systematic search was computed in PubMed/MEDLINE and the Cochrane Library and 3649 potentially relevant papers were detected and screened (n = 3364 following the removal of duplicates). After the removal of irrelevant manuscripts based on the screening of their titles and abstracts (n = 3037), we examined the full-texts of 327 original papers. Finally, after we applied the exclusion and inclusion criteria, a number of 124 original articles were included in this review. Overall, the data analyzed in this review point out an association of Mg concentrations in the body with serum lipids in dyslipidemia and related disorders. However, further research is warranted to clarify whether a higher intake of Mg from the diet or via supplements can influence the lipid profile and exert lipid-lowering actions.

## 1. Introduction

Dyslipidemia has emerged as a significant threat to public health worldwide, with recent statistics revealing that its prevalence reaches 42.7% in China and 56.8% in the United States of America (USA). In addition, Lu et al. (2018) have pointed out that an alarming rate of the population suffering from this disorder is not aware of its lipid profile (26.7% in the USA versus 80.4% in China), leading to poor treatment and control rates of lipid abnormalities (13.2% and 4.6% in China versus 54.1 and 35.7% in the USA) [1]. Thus, identifying novel strategies to combat dyslipidemia are warranted, particularly due to its involvement in the development of and crosstalk with metabolic syndrome (MetS), type 2 diabetes mellitus (T2DM), cardiovascular disease, obesity, hypertension, chronic kidney disease (CKD), and others [1,2,3].

Magnesium (Mg) seems to play a key role in a myriad of disorders, e.g., MetS, T2DM, obesity, hypertension, and its deficiency has been regarded as highly prevalent, with Piuri et al. (2021) ranking it as the most common electrolyte imbalance in high-income countries [4,5,6]. Taking this information into consideration, we may hypothesize that there is a crosstalk between Mg and serum lipids which may impact on the pathogenesis of dyslipidemia and its associated comorbidities, as well as that Mg supplementation might provide health benefits in patients suffering from cardiometabolic disorders.

Therefore, the aim of this review is to explore the associations of Mg (dietary intake, Mg concentrations in the body) and the lipid profile, i.e., total cholesterol (TC), triglycerides (TG), high-density lipoprotein cholesterol (HDL-C), low-density lipoprotein cholesterol (LDL-C), other lipoproteins (Lp), or apolipoproteins (apo), as well as the impact of Mg supplementation on these variables based on data derived from high-quality evidence such as randomized clinical trials (RCTs). 

## 2. Materials and Methods

The protocol employed in this systematic review was based on the Preferred Reporting Items for Systematic reviews and Meta-Analyses (PRISMA) checklist [7].

Four investigators (M.-A.G., E.-C.D., M.-A.C., and N.-I.A.) independently computed a literature search in PubMed/MEDLINE and Cochrane Library from the inception of these databases until 25 February 2021. The following keywords and combinations of words was employed: (“magnesium” OR “magnesemia*” OR “magnesaemia”) AND (“Lp(a)” OR “Triglycerides” OR “Cholesterol” OR “Cholesterol” OR “Lipoproteins, HDL” OR “Cholesterol, HDL” OR “Cholesterol, LDL” OR “Lipoproteins, LDL” OR “Hyperlipidemias” OR “Dyslipidemias” OR “Hypercholesterolemia” OR “lipoprotein triglyceride” OR LDL OR HDL OR “Total cholesterol” OR TG OR Triglyceride OR Triacylglycerol OR TAG OR “lipid profile” OR “low density lipoprotein” OR “high density lipoprotein” OR “blood lipids” OR “lipids*” OR “triglycerid*” OR “trigly*” OR triacylglycerol OR cholesterol OR LDL-C OR HDL-C OR Hyperlipidemia OR Hyperlipidemic OR Dyslipidemia OR Dyslipidemic OR Hypercholesterolemia OR Hypercholesterolaemia OR Hypercholesterolemic OR hypercholesterolaemic) NOT (review OR mice OR rats OR rodents).

We decided for the following inclusion criteria: 1. Original articles/research letters evaluating the relationship between Mg and serum lipids in dyslipidemia and related disorders OR Original articles/research letters evaluating the effects of Mg intake/supplementation on serum lipids in dyslipidemia and related disorders in humans; 2. the subjects recruited in these original studies were adults (aged ≥18 years); 3. the papers were published in English, French, Italian, or Romanian (the languages spoken by the investigators); 4. the papers provided sufficient data regarding the relationship of Mg and serum lipids or the effects of Mg supplementation on serum lipids in dyslipidemia and related disorders; 5. the full-text of the papers could be downloaded/retrieved. We decided for the following exclusion criteria: 1. Reviews, letters to the editor, case reports, conference abstracts, grey literature; 2. the studies were conducted in vitro, on animals or in human subjects aged <18 years; 3. the papers were published in languages unknown to the authors (e.g., Chinese, Polish etc.); 4. the papers did not report sufficient data on the outcomes; 5. the full-text of the articles was unavailable to the investigators. 

Relevant data were extracted independently by four investigators (M.-A.G., E.-C.D., M.-A.C., and N.-I.A.) and disagreements were resolved by consultation with the senior authors (C.C.D. and A.M.G.). 

## 3. Results

**Figure 1:**
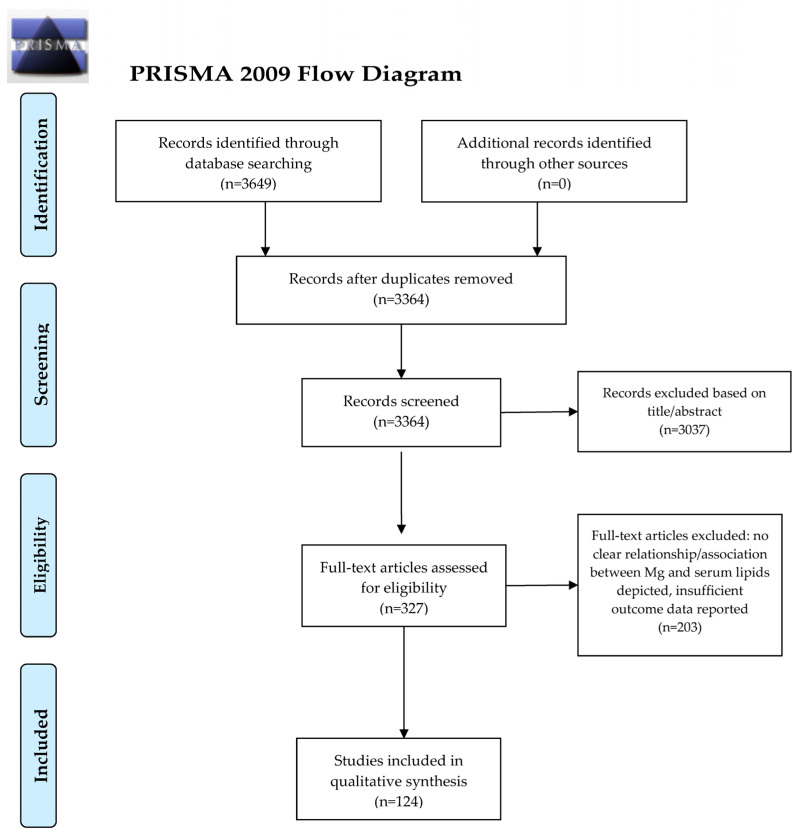
PRISMA 2009 Flow Diagram. From Moher, D.; Liberati, A.; Tetzlaff, J.; Altman, D.G. The Prisma Group. Preferred reporting items for systematic reviews and meta-analyses: The PRISMA statement. *PLoS Med*. **2009**, *6*, e1000097, doi:10.1371/journal.pmed.1000097. For more information, visit www.prisma-statement.org (accessed on 18 March 2021) [7].

A total of 3649 potentially relevant papers were detected and screened. A flowchart diagram of the detailed steps of the literature search process is illustrated in Figure 1. After we removed the duplicates and excluded the irrelevant manuscripts based on the screening of their titles and abstracts (n = 3322), we examined the full-texts of 327 original papers. Finally, after we applied the exclusion and inclusion criteria, a number of 124 original articles were included in this review.

### 3.1. Crosstalk of Magnesium, Serum Lipids, and Dyslipidemia

A total of 36 studies assessed the crosstalk of Mg, serum lipids, and dyslipidemia, of which the vast majority was focused on the relationship of serum Mg with the lipid profile and lipid abnormalities, including the impact of dietary Mg intake or Mg supplementation on these parameters (n = 25). Several studies tackled the Mg-serum lipids crosstalk in overweight/obesity, polycystic ovary syndrome, or nonalcoholic fatty liver disease (n = 11). The most relevant information of this subsection are summarized in Table 1. 

#### 3.1.1. Magnesium, Serum Lipids, and Dyslipidemia

Barragán et al. (2020) evaluated the crosstalk between Mg concentrations, hypercholesterolemia, T2DM, and other cardiovascular risk factors in a Spanish cohort of 492 subjects. Hypomagnesemia was detected in nearly 19% of the study group, with no significant sex-differences (*p* = 0.663), including in terms of plasma Mg levels (*p* = 0.106). Females had higher TC (*p* = 0.006) and HDL-C (*p* < 0.001), lower TG (*p* < 0.001), similar LDL-C (*p* = 0.781) versus men. Men were more likely to suffer from obesity (*p* = 0.012), T2DM (*p* = 0.039), hypertension (*p* < 0.001), use lipid-lowering (*p* = 0.040) or blood pressure-lowering drugs (*p* < 0.001) versus females, but the prevalence of hypercholesterolemia was similar (*p* = 0.186). Mg concentrations were increased in the plasma of hypercholesteremic subjects (*p* = 0.001) and decreased in the plasma of diabetic subjects (*p* = 0.009) as compared to individuals without hypercholesterolemia or T2DM, respectively. There was a significant association between T2DM but not hypercholesterolemia and hypomagnesemia (OR: 3.36, 95% CI: 1.26–8.96, *p* = 0.016 and OR: 1.38, 95% CI: 0.81–2.35, *p* = 0.233). However, there was an association between TC (*p* = 0.01) and LDL-C (*p* = 0.002), but HDL-C (*p* = 0.933) or TG (0.959), and plasma Mg quartiles. Furthermore, patients in the fourth versus first quartile of Mg concentrations were more likely to be diagnosed with hypercholesterolemia (OR: 3.12; 95% CI: 1.66–5.85, *p* < 0.001) [8]. Bersohn and Oelofse (1957) examined a number of forty seven healthy European and fifty three healthy African Bantu subjects. They revealed that the individuals with normal TC values had normal Mg values and the subjects who had a lower than normal TC had significantly higher Mg levels. Patients diagnosed with hypercholesterolemia presented significantly lower Mg values as compared to the healthy population [9]. Petersen et al. (1976) evaluated the 6-week intake of 3 g/day of Mg oxide in a group of seventeen patients suffering from hypercholesterolemia and/or hypertriglyceridemia in order to determine its effects on TC and TG. No significant relationship was registered between the initial values of serum Mg and TG (r = 0.05, *p* > 0.05) or TC (r = −0.21, *p* > 0.05). The authors discharged the hypothesis that 3 g/day of Mg has TC- or TG-lowering actions [10]. In a cross-sectional study, Liu et al. (2020) analyzed the relationship between Mg concentrations and dyslipidemia. Patients diagnosed with dyslipidemia had higher TC, TG, HDL-C, and LDL-C (*p* < 0.001) and lower Mg levels (*p* = 0.002) versus the subjects with normal serum lipids. Subjects in the fourth versus first quartile of serum Mg had an increased risk of dyslipidemia (OR = 1.4, 95% CI 1.0–1.9, *p* = 0.023) in the unadjusted model, but this finding was not verified in the adjusted model [11].

#### 3.1.2. Magnesium, Serum Lipids, and the Diet/dietary Interventions

In a retrospective analysis, Jin and Nicodemus-Johnson (2018) scrutinized 12,284 individuals from the United States’ 2001–2013 National Health and Nutrition Examination Study (NHANES) and observed that, in females, Mg intake was positively associated with HDL-C levels, while it was negatively associated with the TC/HDL-C ratio. However, TG levels were negatively correlated with Mg intake in both genders [12]. Itoh et al. (1997) executed an RCT in which they administered Mg in 33 healthy Japanese subjects, discovering an increase in HDL-C and apoA1, as well as a significant decrease in LDL-C in the Mg group [13]. In Marken et al. (1989)’s RCT, the administration of 400 mg Mg oxide for 60 days to 50 healthy volunteers did not result in changes of TC, HDL-C, LDL-C, VLDL, or TG versus placebo [14]. According to Randell et al. (2008), a number of 1318 healthy individuals recruited in their study displayed significant positive correlations between Mg and TC, HDL-C, LDL-C, and TG [15]. In a cross-sectional study that administered Mg and potassium at variable doses in 529 healthy individuals, Guerrero-Romero et al. (2019) showed significant TG decreases (*p* < 0.0005) and no HDL-C alterations in the subjects receiving recommended versus suboptimal Mg doses [16]. De Valk et al. investigated the link between serum Mg variations and lipolysis-induced TG generation, revealing that an elevation in serum Mg was parallel by an elevation in TG as well (*p* < 0.001) [17]. Aslanabadi et al. (2014) explored the lipid-lowering effects of 1 daily liter of mineral-rich versus normal mineral water in an RCT conducted in 69 adults diagnosed with dyslipidemia (intervention group: 32 subjects, control group: 37 subjects). Despite the fact that the beverage which was enriched with Mg, calcium, sulfate, and bicarbonate lowered TC and LDL-C, the same outcome was reported in the control group as well and there was no statistical difference between the results. Both waters failed to exhibit an impact of TG or HDL-C [18]. According to Fu et al. (2012), the consumption of deep sea water (395 Mg mg/L) versus MgCl_2_ fortified (386 Mg mg/L) or reverse osmotic water decreased TC, LDL-C, and HDL-C in a time-dependent manner [19]. Based on Nerbrand et al. (2003)’s research, there are no correlations between the content of Mg in the water and cardiovascular risk factors [20]. Luoma et al. (1973) studied the relationship of Mg concentrations in the drinking water with several cardiovascular risk factors (including TG and TC) in 300 men from four different Finnish rural districts, but found no association between these variables [21]. Low-energy dietary interventions which also involved Mg supplementation achieved reductions in TC, LDL-C, and TG, but also decreased HDL-C, according to a pre-post intervention evaluation of 49 subjects from the United States [22]. In de Los Rios (1963)’s research project, a number of twenty eight schizophrenic patients were prescribed a strictly controlled diet which included Mg in quantities above the recommended dietary values, but this nutrition experiment found no association between the alterations of Mg levels and TC [23]. A higher dietary consumption of Mg seems to be linked with oxidized LDL, a biomarker of oxidative stress, as concluded by Cocate et al. (2013) who assessed the intake of vegetables, fruit, and nutrients of 296 middle-aged males with a normal status of health [24]. Patients with multimorbidity (including hypercholesterolemia) are known to have smaller daily intakes of Mg, whereas an increased consumption of cereals (r = 0.60, *p* < 0.0001) and fruits and vegetables (r = 0.49, *p* < 0.0001) were associated with higher serum Mg levels and less multimorbidity [25]. There seems to be an inverse correlation between the intake of Mg and TC in both males (*p* = 0.02) and females (*p* = 0.04), according to Bain et al. (2015) who evaluated 4443 subjects aged 40–75 from the European Prospective Investigation into Cancer)-Norfolk cohort in order to assess the relationship between the risk of stroke and its most important risk factors and the dietary consumption of Mg [26]. In addition, Samavarchi Tehrani et al. (2020) recorded a significant association (*p* = 0.012) between the presence of dyslipidemia and Mg levels in 447 patients suffering a stroke of embolic origin [27]. Interestingly, Kim et al. (2014), based on best-fit models from stepwise linear regressions, discovered that the dietary intake of Mg predicts the concentrations of HDL-C and its subspecies. In their study group of 1566 individuals, the dietary consumption of Mg shared positive associations with HDL-C (coefficient ± SE: 4.79 ± 1.45; %HDL-C variation: 0.12%, *p* = 0.001) and its subfractions, HDL-2 (coefficient ± SE: 1.43 ± 0.61; %HDL-C variation: 0.028%, *p* = 0.018) and HDL-3 (coefficient ± SE: 2.98 ± 1.20; %HDL-C variation: 0.085%, *p* = 0.013), but not with apoA1 concentrations [28]. Kim and Choi (2013) investigated the dietary intake of Mg and its relationship with the lipid profile in 258 healthy Korean adults. Although men had a higher Mg daily consumption versus women, they registered higher TG (*p* < 0.05) and lower HDL-C (*p* < 0.01) values, yet similar TC and LDL-C levels. The atherogenic index of plasma was also elevated in males versus females (*p* < 0.001). However, the correlations between serum Mg and TC, HDL-C, LDL-C, TG or the atherogenic index of plasma did not reach statistical significance [29]. Cao et al. (2015) analyzed the relationships between serum and urinary Mg concentrations and the lipid profile in 2837 middle-aged/elderly Chinese, revealing a positive association between higher serum Mg and TC (*p* < 0.001), HDL-C (*p* < 0.001), LDL-C (*p* = 0.001), and TG (*p* < 0.001), and negative one with non-HDL-C/HDL-C (*p* = 0.003). There were also positive associations between the Mg/creatinine ratio in the urine and TC (*p* = 0.004), HDL-C (*p* = 0.003), and LDL-C (*p* = 0.009). However, there were some gender-based differences regarding these results: in males, the associations between serum Mg and LDL-C, TG or non-HDL-C/HDL-C, and those between urinary Mg/creatinine and HDL-C or LDL-C, were not statistically significant [30]. On the other hand, there are atomic absorption spectrophotometry studies conducted in postmenopausal females that show that Mg concentrations in red blood cells and not serum Mg concentrations correlate with serum TG (r = 0.287, *p* = 0.011) [31]. Data derived from the Cardiovascular Disease and Alimentary Comparison (CARDIAC) study also reinforced that hypercholesterolemia was more prevalent and TC concentrations were higher in individuals with lower versus higher 24-h urinary Mg/creatinine ratios (*p* < 0.001 for trend for both). Subjects in the lowest quintiles of 24-h Mg/creatinine urinary ratios were more likely to suffer from hypercholesterolemia (OR = 2.73; 95% CI 2.03 to 3.67; *p* < 0.001) versus the highest quintiles [32]. 

#### 3.1.3. Magnesium, Serum Lipids, and Overweight and (or) Obesity

Guerrero-Romero and Rodriguez-Moran (2013) investigated the relationship between serum Mg and several metabolic phenotypes, namely healthy normal-weight (NW) versus metabolically obese normal weight (MONW) subjects, as well as obese versus metabolically healthy obese (MHO) patients. NW and MHO subjects exhibited higher serum Mg concentrations (*p* = 0.04 and *p* = 0.01, respectively) and lower TG (*p* < 0.0005 for both), yet similar HDL-C levels, versus their corresponding comparators. In MONW patients, there was a negative correlation between TG and serum Mg (r = −0.530, unreported *p*-value). Moreover, in both obese and in particular non-obese patients, low serum Mg concentrations were associated with the presence of hypertriglyceridemia (OR = 1.61, 95% CI: 1.5–2.46 and OR = 6.67, 95% CI 2.1–20.4, respectively) [33]. However, Mg levels did not correlate with TG concentrations in another study on obese individuals who were planning to undergo bariatric surgery [34]. In Rodriguez-Moran and Guerrero-Romero (2014)’s RCT, the 4-month daily administration of 30 mL of MgCl_2_ 5% solution (equivalent to 382 mg of Mg) was compared to the administration of 30 mL of placebo solution in hypomagnesemic MONW subjects. Following the intervention, HDL-C increased (*p* < 0.05) and TG levels decreased (*p* < 0.0001) significantly [35]. In their RCT, Joris et al. (2017) examined the effect of long-term Mg supplementation on endothelial function and multiple cardiometabolic risk markers in subjects suffering from overweight or obesity. Fifty two subjects were assigned randomly to two different groups that received either a dose consisting of 350 mg Mg or placebo, yet no differences in TC, HDL-C, LDL-C, TG or non-esterified fatty acids were observed in the intervention versus control groups [36]. Guerrero-Romero et al. (2016) investigated the relationship between obesity and hypomagnesemia in six hundred and eighty one subjects. When separating the subjects based on their Mg levels, their findings reported a significant difference in the mean values of HDL-C (1 ± 0.3 mmol/L in the low serum Mg group and 1.2 ± 0.4 mmol/L in the normal serum Mg group), as well as in TG (1.9 ± 1.4 mmol/L in the low serum Mg group and 1.8 ± 1.5 mmol/L in the normal serum Mg group) [37]. Solati et al. (2019) executed a 6-month RCT in which they administered 300 mg Mg sulfate in the form of herbal supplements versus placebo in overweight subjects who did not suffer from T2DM. Mg administration increased HDL-C (*p* < 0.001) and HDL-C/TG (*p* < 0.0001) and lowered LDL-C (*p* < 0.05) and TG (*p* < 0.05) values [38].

#### 3.1.4. Magnesium, Serum Lipids, and Polycystic Ovary Syndrome (PCOS)

Mg supplementation (250 mg/day Mg oxide for 8 weeks) in females with polycystic ovary syndrome did not lead to significant changes in TC, HDL-C, LDL-C, TG, TC/HDL-C, or TG/HDL-C versus placebo [39]. Cutler et al. (2019) inspected the nutritional intakes of 87 women with PCOS and reported that females following a Mg-rich diet had less insulin resistance (*p* = 0.02) and higher HDL-C (*p* = 0.02) [40]. In Jamilian et al. (2019)’s RCT, 60 women with PCOS who received Mg+vitamin E displayed significant reductions in TG (*p* = 0.001) and VLDL (*p* = 0.01), but no alterations in LDL-C, HDL-C, or TC/HDL-C [41]. Moreover, in another RCT, Jamilian et al. (2017), TG (*p* < 0.001), VLDL (*p* < 0.001), and TC (*p* = 0.04) decreased significantly, but significant effect on LDL-C or HDL-C was seen in the 60 women with PCOS that received Mg, zinc, calcium, and vitamin D co-supplementation [42].

#### 3.1.5. Magnesium, Serum Lipids, and Nonalcoholic Fatty Liver Disease (NAFLD)

Karandish et al. (2013) conducted an RCT in 34 NAFLD subjects versus 34 healthy controls in which they investigated the benefits of Mg supplementation in combination with a low-calorie diet and physical exercise on several biochemical parameters. NAFDL patients who received the intervention consisting in supplementation with Mg did not experience statistically significant alterations of TC, LDL-C, HDL-C, or TG concentrations, whereas LDL-C (*p* = 0.000) and TC (*p* = 0.003) concentrations dropped significantly in the control group who did not receive Mg and only practiced physical exercise and caloric restriction [43].

### 3.2. Crosstalk of Magnesium, Serum Lipids, and Metabolic Syndrome

A total of 16 studies assessed the crosstalk of Mg, serum lipids, and MetS, focusing on the relationship of serum Mg with the lipid profile in this metabolic disorder, including the impact of dietary Mg intake or Mg supplementation on serum lipids.

Ali et al. (2013) investigated the dietary intakes of 213 American Indians diagnosed with MetS who were enrolled into the Balance Study RCT. The authors detected that nearly 90% of the male subjects had Mg intakes below the daily recommended dose, mainly due to dietary patterns poor in whole grains and vegetables which are sources of Mg [44]. Similarly, Vajdi et al. (2020) reported that subjects who follow a nutrient pattern based on plant sources which is also rich in Mg depict lower odds of MetS (*p* = 0.01) and lower LDL-C (*p* = 0.04), but similar TC, TG, and HDL-C (*p* > 0.05 for all) when comparing the first to the fourth quartile of this diet [45]. However, Akbarzade et al. (2020) did not detect an association between a nutrient pattern which comprised a higher Mg intake and MetS components in Iranian adults [46]. Similarly, Mottaghian et al. (2020) did not discover any association between the Mg-rich dietary pattern and alterations of the serum lipids during a 3-year timeframe. Nevertheless, TG increased (*p* < 0.05 for trend) across the quartiles of the Mg-rich nutrient pattern [47]. Choi and Bae (2013) evaluated the intake of Mg and the risk of MetS in Korean adults, revealing that both males and females diagnosed with MetS had lower Mg intakes and percentages of the recommended nutrient intake of intake versus healthy controls. Moreover, nearly a half of the MetS subjects did not achieve the estimated average requirement of Mg. However, the authors did not detect a link between the risk of MetS and the intake of Mg after adjusting for potential confounders. Women with low HDL-C concentrations had a lower Mg consumption, yet the same finding was not verified in men. In both genders, higher TG levels could not be linked to Mg intakes [48]. Cano-Ibáñez et al. (2019) investigated the relationship between MetS and Mg concentrations in 6646 individuals and discovered that subjects with MetS have a lower dietary Mg intake. Low levels of education and male sex were linked with smaller dietary intakes of this nutrient [49]. Choi et al. (2014) detected no differences between MetS subjects and healthy controls in terms of serum Mg levels. However, when analyzing the hair mineral concentrations, they detected lower Mg concentrations (*p* = 0.046) and a higher sodium/magnesium ratio (*p* = 0.013) in MetS patients. Moreover, there was negative significant correlation between hair Mg and TG (r = −0.125, *p* < 0.05) and positive non-significant Mg-HDL-C correlation (r = 0.093, *p* > 0.05) [50]. Vanaelst et al. (2012) also analyzed hair Mg levels and depicted negative correlations between serum Mg and non-HDL-C (r = −0.170, *p* = 0.030) and metabolic score (r = −0.257, *p* = 0.001) in Belgian schoolgirls. Hair Mg concentrations were significantly lower (*p* = 0.015) in females with a metabolic score of more than 3 points [51]. However, Sun et al. (2013) detected lower Mg (*p* < 0.001) and HDL-C and higher TC, TG, and LDL-C levels in Chinese subjects with MetS versus controls [52]. Despite low serum and intramononuclear levels of Mg in non-diabetic females diagnosed with MetS, de Lourdes Lima de Souza e Silva (2014) did not register any changes in TG, TC, HDL-C, or LDL-C following supplementation with 400 mg of Mg chelate versus placebo in their 12-week RCT [53]. Rotter et al. (2015) evaluated the concentrations of heavy metals and bioelements in 313 Polish men aged 50–75 years and detected lower Mg levels in patients diagnosed with MetS (*p* = 0.02), T2DM (*p* = 0.0001), and hypertension (*p* = 0.0001). Overweight/obese and normal-weight individuals had similar Mg concentrations (*p* = 0.41). The authors depicted positive associations between Mg and TC (r = 0.25; *p* < 0.001) and LDL-C (r = 0.26; *p* < 0.001), however the Mg-HDL-C or Mg-TG correlations did not reach statistical significance [54]. Ghasemi et al. (2010) retrospectively analyzed 137 individuals aged >60 years and unmasked that patients with MetS, T2DM and hyperglycemia depict lower Mg levels [55]. Evangelopoulos et al. (2008) also exposed that Mg was positively correlated with HDL-C (r = 0.18; *p* = 0.05) in patients affected by MetS [56]. In a population-based research (192 MetS subjects versus 384 healthy controls), Guerrero-Romero and Rodríguez-Morán (2002) observed a strong connection of MetS dyslipidemia and Mg deficiency based on the Mg-HDL-C (r = 0.36, *p* < 0.05), Mg-TC (r = −0.29, *p* < 0.05) associations [57]. Yuan et al. (2016) found multiple statistically significant correlations between serum Mg, calcium, Ca/Mg levels and metabolic risk factors for MetS. Their study included two hundred and four MetS patients and two hundred and four healthy subjects as the control group. Multiple blood tests were performed and the values of serum Mg and blood lipids were determined. Correlation studies were performed and the following results were described: positive correlation between serum Mg and BMI (r = 0.128, *p* < 0.05), TC (r = 0.254, *p* < 0.05), and LDL-C (r = 0.280, *p* < 0.05) [58]. Rotter et al. (2016) observed statistically significant, positive correlations between Mg and TC (r = 0.25, *p* < 0.0001) and LDL-C (r = 0.26, *p* < 0.0001). Their results found no statistically significant correlations between Mg and HDL-C (r = 0.009, *p* = 0.87) or TG (r = −0.06, *p* = 0.28). Three hundred and thirteen men were involved in their research which had the objective of determining the relationship between serum Mg concentrations and the occurrence of metabolic and/or hormonal disorders [59].

The most relevant information of this subsection are summarized in Table 2.

### 3.3. Magnesium, Serum Lipids, and Type 2 Diabetes Mellitus (T2DM)

A total of 40 studies assessed the crosstalk of Mg, serum lipids, and T2DM, focusing on the relationship of serum Mg with the lipid profile in this metabolic disorder, including the impact of dietary Mg intake or Mg supplementation on serum lipids. The associations of hypomagnesemia with the lipid profile was evaluated in eight papers, whereas the benefits of Mg supplementation (data mostly derived from RCTs) was scrutinized in 21 manuscripts. 

Based on data from 5568 subjects enrolled in the Prevention of Renal and Vascular End-stage Disease (PREVEND) study, van Dijk et al. (2019) unveiled that Mg levels (assessed by both nuclear magnetic resonance spectroscopy and colorimetric assays) are lower in T2DM (*p* < 0.001 for both methods). In the entire study population, there was a negative correlation between Mg (measured by nuclear magnetic resonance spectroscopy but not colorimetric assays) and TG (r = −0.073, *p* < 0.001 and r ≤ 0.001, *p* = 0.99, respectively). In T2DM subjects, there was a negative correlation of Mg (measured by nuclear magnetic resonance spectroscopy and colorimetric assays) and TG (r = −0.184, *p* = 0.002 and r = −0.194, *p* = 0.001, respectively). In the entire study population, according to the results of the multivariable linear regression, there was a correlation between Mg (measured by nuclear magnetic resonance spectroscopy) and low HDL-C concentrations (β = −0.062, *p* < 0.001), but not with high TG concentrations (β = −0.011, *p* = 0.45). There was no association between Mg levels measured by colorimetric assays and HDL-C or TG [60]. As compared to healthy controls, Rusu et al. (2013) demonstrated that T2DM patients, and particularly those suffering from peripheral arterial disease, have lower Mg (*p* < 0.01) and HDL-C and higher TG and TC levels [61]. Spiga et al. (2019) evaluated the associations of Mg concentrations and serum lipids in patients with impaired fasting glucose or T2DM. Mg and HDL-C levels decreased and TG increased as follows: patients with normal glucose tolerance → impaired fasting glucose → T2DM (*p* < 0.02, *p* < 0.01 and *p* < 0.001, respectively). Significant correlations of Mg with TC (r = 0.154, *p* < 0.001), HDL-C (r = 0.113, *p* < 0.01), LDL-C (r = 0.170, *p* < 0.001), but not with TG (r = 0.01, *p* = 0.981). Higher Mg concentrations were linked with a lower risk of T2DM (OR = 0.765, 95% CI 0.629–0.932, *p* < 0.01), including in the non-diabetic subjects who were followed-up for nearly 6 years (HR = 0.790, 95% CI: 0.645–0.967; *p* = 0.022) [62]. Esmeralda et al. (2021) investigated the link between TC, TG and serum/urinary Mg in T2DM versus healthy counterparts. T2DM subjects had higher TG (*p* = 0.004) and fractional excretion of Mg (*p* = 0.01), lower serum Mg (*p* = 0.001) and similar TC (*p* = 0.31) and urinary Mg (*p* = 0.097) versus controls. Nevertheless, no associations of the serum Mg or the fractional excretion of Mg with TC or TG were detected [63]. Gopal et al. (2019) concluded that serum Mg concentrations predict the development of proliferative retinopathy in patients with T2DM (optimum cut-off 1.7 mg/dL, sensitivity 92.86%, specificity 77.14%, AUC 0.837, SEM 0.06, 95% CI 0.70–0.92). In T2DM, Mg levels decreased as following no retinopathy → non-proliferative retinopathy → proliferative retinopathy (*p* < 0.01). Surprisingly, patients with proliferative retinopathy had higher HDL-C values (*p* < 0.05). TC, TG, LDL-C, and VLDL concentrations did not differ significantly among the study groups [64]. Hruby et al. (2017) studied the link between the intake of Mg and the risk of T2DM in three cohorts from the United States of America, detecting a 15% T2DM-risk reduction in individuals with a higher dietary intake of Mg. Hypercholesterolemia was more frequent in the fifth (12.7% for 427–498 mg/day) versus first (8.7% for 242–275 mg/day) quartile of Mg intake, whereas in women the data were conflicting: in one cohort, hypercholesterolemia was more prevalent in the fifth (7.8% for 357–418 mg/day) versus first (6.2% for 187–218 mg/day) quartile of Mg intake, whereas in the other cohort the results were opposite (15.4% for 213–245 mg/day versus 14.4% for 385–448 mg/day) [65]. In Anetor et al. (2002)’s research on 40 T2DM patients from Nigeria, only TC displayed a significant positive correlation with Mg levels (r = 0.6; *p* < 0.001) [66]. Corica et al. (2006) analyzed 290 T2DM and detected that serum Mg was significantly lower in individuals with low HDL-C (*p* < 0.001) and high TG (*p* < 0.001) [67]. Romero and Moran (2000) evaluated 180 subjects with impaired glucose regulation (un/controlled T2DM and IFG) versus 190 healthy controls and demonstrated that decreased Mg levels are associated with decreased HDL-C, regardless of blood glucose values (*p* = 0.01 for the T2DM groups, *p* = 0.05 for the IFG group and *p* = 0.03 for the control group) [68]. Yu et al. (2018) reported that a group of 8163 Chinese T2DM adults, classified based on Mg levels, exhibited significant elevations in serum lipids, except for HDL-C, across progressive concentrations of serum Mg (from low Mg levels of ≤0.65 mmol/L normal levels 0.65–0.95 mmol/L, high levels ≥0.95 mmol/L) (*p* < 0.05), regardless of whether they suffered from central obesity or not. The generalized linear model showed that after the full adjustment for demographic characteristics, lifestyle, dietary, and clinical factors, TG, TC, HDL-C, and LDL-C were significantly higher in subjects with Mg levels ≥0.95 mmol/L versus those with lower Mg levels (*p* < 0.05) [69]. Kurstjens et al. (2016) investigated the determinants of serum Mg levels in a T2DM cohort (n = 395). Multiple blood parameters were investigated including serum Mg levels, HDL-C, LDL-C, TC, TG, and others, and correlation studies were performed. Their results showed a statistically significant negative correlation between TG and serum Mg (r = −0.273, *p* < 0.001), as well as a positive correlation between HDL-C and Mg (r = 0.156, *p* = 0.002) [70].

The most relevant information of this subsection are summarized in Table 3. 

#### 3.3.1. Hypomagnesemia

Rasheed et al. (2012) compared laboratory variables in 219 T2DM patients and 100 healthy controls, depicting a higher prevalence of hypomagnesemia, higher TG and LDL-C and lower HDL-C concentrations in the study group [71]. Srinivasan el al. (2012) also unmasked that, in T2DM, hypomagnesemia not normomagnesemia positively correlated with TG concentrations (*p* < 0.05) [72]. Pokharel et al. (2017) researched the association of serum Mg and different cardiovascular risk factors in 150 Nepalese T2DM subjects versus 50 controls. Hypomagnesemia was discovered in 50% of T2DM individuals versus 0% in the healthy counterparts. An inverse correlation between serum Mg and TC (r = −0.219; *p* < 0.01) and LDL-C (r = −0.168; *p* < 0.05) was detected. Mg concentrations were similar (*p* > 0.05) between overweight, hypertensive, and dyslipidemic T2DM patients and the control group. The authors concluded that based on their study a nutritional supplement of Mg was warranted for the prevention and minimization of chronic T2DM systemic complications such as insulin resistance and dyslipidemia [73]. Hyassat et al. (2014) analyzed 1105 overweight/obese subjects diagnosed with T2DM and detected a prevalence of hypomagnesemia of 19% (95% CI: 16.8–21.4%) in the study group. Patients who were dyslipidemic or were prescribed statins were more likely to associate low serum Mg concentrations (*p* = 0.022 and *p* < 0.001, respectively). Low serum Mg levels remained associated with the administration of statins in the multivariate logistic regression as well (OR = 1.56, 95% CI: 1.1–2.2) [74]. Waanders et al. (2019) investigated the crosstalk between T2DM and hypomagnesemia in 929 Dutch individuals, demonstrating that Mg concentrations correlated with TC (r = 0.142, *p* < 0.001), TC/HDL-C ratio (r = 0.114, *p* < 0.001), and LDL-C (r = 0.166, *p* < 0.001), but not with HDL-C (r = −0.003, *p* = 0.923) or TG (r = 0.002, *p* = 0.941). The results of the stepwise multivariable regression revealed a significant association of serum Mg with LDL-C (β = 0.141, *p* = 0.001) [75]. In Huang et al. (2012)’s cross-sectional observational study on 210 T2DM subjects aged >65 years, the appraisal of nutritional habits unmasked an insufficient Mg intake in >88% and hypomagnesemia in 37% of the individuals. Mg intake was positively correlated with HDL-C (r = 0.192; *p* = 0.005) [76]. Corsonello et al. (2000) conducted a study on 90 T2DM patients (30 patients without albuminuria, 30 with microalbuminuria and 30 with proteinuria) and observed a decrease in serum Mg and an increase in TG as the urinary protein loss increased (*p* < 0.001). However, the presence of hypomagnesemia was not associated with hypercholesterolemia, despite higher TC being detected in the patients with hypomagnesemia [77]. Shardha et al. (2014) investigated the relationship between serum Mg levels and serum lipids in T2DM individuals suffering from hypokalemia. TG (234.50 mg/dl versus 169 mg/dL; *p* = 0.001) and LDL-C (123 mg/dL versus 105 mg/dL; *p* < 0.001) were higher and HDL-C (39 mg/dL versus 46 mg/dL; *p* < 0.001) was lower in hypokalemic T2DM individuals who associated hypomagnesemia [78].

The most relevant information of this subsection are summarized in Table 4. 

#### 3.3.2. Magnesium Supplementation in RCTs and Interventional Studies

Song et al. (2006)’s meta-analysis of RCTs unmasked that supplementation with Mg elevated HDL-C in T2DM subjects (+0.08 mmol/L, 95% CI 0.03–0.14, *p* = 0.36 for heterogeneity), but failed to produce effects on TG, TC or LDL-C [79]. Hamedifard et al. (2020) conducted a 12-week RCT in which they compared the administration of 250 mg Mg oxide plus 150 mg zinc sulfate versus placebo in 60 T2DM subjects suffering from coronary heart disease. The intervention did not yield any significant changes in TC, TC/HDL-C ratio, LDL-C, VLDL or TG, however it resulted in a notable increase in HDL-C (β = 2.09 mg/dL, 95% CI, 0.05, 4.13; *p* = 0.04) versus placebo [80]. Al-Daghri et al. (2014) supplemented a cohort of 126 Saudi T2DM subjects with vitamin D and detected a significant positive correlation between serum Mg and TG (r = 0.32, *p* = 0.04) in males. In females, there was a positive correlation (r = 0.36, *p* = 0.006) between HDL-C and the variation in Mg concentrations following 6 months of vitamin D supplementation [81]. Rashvand et al. (2019) explored the impact of 500 mg Mg oxide versus 1000 mg choline bitartrate versus Mg + choline co-supplementation versus placebo on the lipid profile of 96 T2DM subjects in a 2-month RCT. Mg supplementation alone raised serum Mg concentrations (*p* = 0.02), but failed to alter the values of TG (*p* = 0.24), TC (*p* = 0.48), LDL-C (*p* = 0.89), HDL-C (*p* = 0.09), or LDL-C/HDL-C (*p* = 0.62). Choline supplementation alone did not have any impact on the lipid profile as well. However, the Mg+choline intervention elevated Mg levels (*p* < 0.001 versus *p* = 0.02 for Mg supplementation alone), decreased TG (*p* = 0.04), increased HDL-C (*p* = 0.01), but did not alter TC (*p* = 0.92), LDL-C (*p* = 0.44) or LDL-C/HDL-C (*p* = 0.08) [82]. Dietary interventions, such as honey enriched with Mg, cinnamon, and chromium, was also able to decrease LDL-C (−0.29 mmol/L; 95%CI −0.57 to −0.23; *p* = 0.039) and TC (−0.37 mmol/L; 95% CI −0.073 to −0.008; *p* = 0.046) values versus standard honey in an RCT involving subjects diagnosed with T2DM, yet TG and HDL-C values remained unaltered [83]. In their recent meta-analysis of 4 RCTs, Dehbalaei et al. (2021) analyzed the effects of combined 250 mg/day of Mg and 400 IU/day of vitamin E supplementation for 6–12 weeks on the lipid profile in 119 participants suffering from T2DM-induced foot ulcers, gestational diabetes or polycystic ovary syndrome versus 118 controls. The intervention yielded a reduction in TC (WMD: −15.89 mg/dL, 95% CI: 24.39, 7.39, *p* < 0.001), LDL-C (WMD: −11.37 mg/dL, 95% CI: 19.32, 3.41, *p* = 0.005), and TG (WMD: −26.97 mg/dL, 95% CI: 46.03, 7.90, *p* = 0.006), but did not influence HDL-C (WMD: 1.59 mg/dL, 95% CI: 0.17, 3.35, *p* = 0.076) [84]. According to Yokota et al. (2004), a 30-day supplementation with 300 mg/day of Mg did not modify TC, HDL-C, or TG in T2DM subjects [85]. Djurhuus et al. (2001) monitored the effects of 24 weeks of oral supplementation with Mg in patients with type 1 diabetes and hypomagnesemia. Although TG levels increased in the first week, at 24 weeks the authors noted a reduction in LDL-C, TC, and apoB. Intravenous administration of Mg resulted in a more pronounced decrease of the same parameters [86]. Ham and Shon (2020) conducted an RCT in which they evaluated the 8-week administration of deep-sea water enriched with Mg versus placebo in 74 individuals with prediabetes. Following the intervention, the authors recorded a significant reduction in LDL-C (*p* = 0.003) and TC (*p* = 0.006), but HDL-C and TG remained unchanged [87]. However, in their RCT, Cosaro et al. (2014) detected no benefits of Mg supplementation in terms of TC, LDL-C, or TG reduction and HDL-C elevation in a small study group of 7 males aged 23–33 years who had a positive family history for T2DM/MetS versus 7 healthy controls [88]. Asemi et al. (2015) conducted an RCT to investigate the effects of Mg supplementation in females suffering from gestational diabetes and concluded that the placebo group had higher values of TC (*p* = 0.01), VLDL (*p* = 0.005), and TG (*p* = 0.005) at the end of the intervention versus the baseline. In both the placebo and the Mg-deficient women’s group, HDL-C, LDL-C and TC/HDL-C ratio did not change significantly throughout the RCT [89]. In Navarrete-Cortes et al. (2014)’s RCT, a 3-month administration of 360 mg Mg daily failed to lead to any changes in TC, TG, LDL-C, or HDL-C in T2DM subjects with normomagnesemia versus placebo [90]. Another RCT, conducted by Guerrero-Romero et al. (2015), explored the benefits of 30 mL of MgCl_2_ 5% solution (equivalent to 382 mg Mg) versus placebo in 59 individuals diagnosed with hypomagnesemia and prediabetes versus controls. At the end of the intervention which lasted for 4 months, the study group displayed an elevation in HDL-C (+4.7 ± 10.5 mg/dL versus −3.9 ± 11.3 mg/dL, *p* = 0.04) and a reduction in TG (−57.1 ± 80.7 mg/dL versus −30.9 ± 87.7, *p* = 0.009) as compared to placebo [91]. In the RCT performed by Solati et al. (2014), 25 T2DM patients who received 300 mg/day of Mg sulfate for a period of 3 months were compared to 22 T2DM patients who received placebo. Following Mg prescription, the intervention group displayed lower LDL-C (93.63 ± 24.58 mg/dL versus 120.4 ± 34.86 mg/dL; *p* < 0.01) and non-HDL-C (125.30 ± 23.19 mg/dL versus 152.16 ± 37.05 mg/dL; *p* < 0.001) levels at the end of the RCT, but TG, TC, and HDL-C concentrations were similar to the placebo group [92]. De Valk et al. (1998) supplemented 34 patients with controlled T2DM with Mg aspartate for 3 months, but failed to detect significant changes in serum lipids in the intervention versus control group [93]. Similarly, the 90-day administration of 600 mg/day of Mg in 56 T2DM patients did not alter TC, HDL-C, or LDL-C [94]. Talari et al. executed an RCT in which 54 Iranian T2DM patients on hemodialysis were prescribed 250 mg/day of Mg oxide for 24 weeks. There was a significant decrease in TC (*p* = 0.02) and LDL-C (*p* = 0.01) in the group receiving Mg [95]. In another 12-week RCT, Afzali et al. (2019) studied the effects of 250 mg/day Mg oxide + 400 IU vitamin E in 57 patients with diabetic foot. The intervention group exhibited a decrease in TG (*p* = 0.04) and LDL-C (*p* = 0.03), as well as an increase in HDL-C (*p* = 0.01) [96]. In a cross-sectional study, Brandao-Lima et al. (2019) researched the impact of zinc, potassium, calcium, and Mg administration at different concentrations in 95 T2DM patients. Higher levels of TG (*p* = 0.01), TC (*p* = 0.097), LDL-C (*p* = 0.0867), and HDL-C (*p* = 0.0247) were observed in the group that received the aforementioned elements in lower concentrations [97]. In a 6-week RCT performed by Karamali M. et al. (2018), 60 patients with gestational diabetes mellitus were given either Mg-zinc-calcium-vitamin D co-supplements or placebo (n = 30 each group). The combined supplementation significantly decreased TG (−0.27 ± 0.89 versus +0.36 ± 0.39 mmol/L, *p* = 0.001) and VLDL (−0.13 ± 0.40 versus +0.16 ± 0.18 mmol/L, *p* = 0.001) as compared to placebo [98]. Sedeghian et al. (2020) conducted a 12-week RCT to assess the effects of Mg sulfate supplementation in 80 patients with early diabetic nephropathy, but did not detect significant changes in serum Mg or TC, LDL-C, HDL-C, TC/HDL-C, or TG [99].

### 3.4. Crosstalk of Magnesium, Serum Lipids and Cardiovascular Disorders 

A total of 16 studies assessed the crosstalk of Mg, serum lipids, and cardiovascular disorders, focusing on the relationship of serum Mg with the lipid profile in atherosclerosis, angina pectoris, acute myocardial infarction, coronary heart disease, coronary artery calcifications, as well as hypertension (n = 7).

#### 3.4.1. Atherosclerosis, Angina Pectoris and Acute Myocardial Infarction

The prevalence of hypercholesterolemia was equally balanced among serum Mg concentrations in 414 patients younger than 50 years old who were subjected to drug-eluding stent implantation following an acute coronary syndrome [100]. Qazmooz et al. (2020) analyzed the crosstalk between several trace elements (including Mg) and subjects with atherosclerosis versus unstable angina versus healthy controls. Patients with atherosclerosis had higher Mg levels versus controls and unstable angina patients. In the subjects diagnosed with atherosclerosis or unstable angina, elevated TC, Castelli index 1 (zTC–zHDL-C) and lower HDL-C levels were recorded as compared to controls. In addition, there were significant differences in terms of LDL-C, TG, atherogenic index of plasma (zTG–zHDL-C), Castelli index 2 (zLDL-C–zHDL-C) between study groups. TG and the atherogenic index of plasma (zTG–zHDL-C) increased as follows: controls → atherosclerosis → unstable angina. LDL-C and the Castelli index 2 (zLDL-C–zHDL-C) increased as follows: controls → unstable angina → atherosclerosis. Based on the results of the multiple regression analysis, Mg was one of the explanatory variables accounting for the variance in the atherogenic index of plasma (β = −0.205, t = −3.036, *p* = 0.003), Castelli index 1 (β = −0.179, t = −2.633, *p* = 0.009), Castelli index 2 (β = −0.143, t = −1.983, *p* = 0.049) and HDL-C (β = 0.157, t = 2.106, *p* = 0.037) [101]. Brown et al. (1958) had also sought to examine the interplay of Mg, serum lipids and myocardial infarction in 186 adults who attended the Cardiovascular Health Center at Albany Medical College annually, but did not discover statistically significant associations of Mg and serum cholesterol, total lipids or alpha/beta lipoproteins [102]. The findings of Mahalle el al. (2012) regarding the crosstalk of Mg and serum lipids in 300 patients with known cardiovascular disease also delineate that TC, LDL-C, VLDL and TG are higher and HDL-C is lower in subjects with low serum Mg [103]. 

#### 3.4.2. Coronary Heart Disease

In the ARIC study, Liao et al. (1998) looked into the correlation between serum Mg levels and risk factors for coronary heart disease, including TC, TG, and HDL-C. The nearly 13,000 patients enrolled exhibited an increase in TC and HDL-C, as well as a decrease in LDL-C, with increasing serum Mg values [104]. Farshidi et al. (2020) conducted a 6-month RCT in which they prescribed 300 mg/day of Mg sulfate to 32 patients with coronary heart disease versus 32 subjects who received placebo. At the 3-month evaluation, the authors demonstrated a decrease in oxLDL and TC/HDL-C ratio versus placebo. Patients with one atherosclerotic vessel benefited from a reduction in LDL-C, whereas patients with more affected vessels registered an elevation in HDL-C. At 6 months, there were significant alterations of oxLDL, LDL-C, and HDL-C in subjects with one interested vessel, as well as LDL-C alterations in patients with at least two atherosclerotic vessels. Notable changes in the concentrations of oxLDL receptors was seen. During the 3 months between evaluations, oxLDL decreased in subjects with only one vessel interested by atherosclerosis [105]. Petersen et al. (1977) examined a cohort of seventy three men and women and evaluated the relationship between serum and erythrocyte Mg and several indicators of coronary heart disease, detecting a significant inverse correlation of serum Mg and systolic blood pressure (r = −0.31, *p* < 0.01), and a significant positive correlation of erythrocyte Mg and TC (r = 0.25, *p* < 0.05) [106].

#### 3.4.3. Coronary Artery Calcifications

Posadas-Sanchez et al. (2016) sought to examine the association between serum Mg levels and coronary artery calcification. The authors included a total of one thousand two hundred and seventy six subjects in their study. Blood serum values of Mg were compared to the values of LDL-C, HDL-C, TG, TC, apolipoproteins A and B, and using regression models, correlation studies were performed. After dividing the study participants according to their serum Mg quartiles, four groups were formed. None of the lipid profile components showed significant differences based on Mg quartiles, with the mean values of LDL-C, HDL-C, TG, apolipoprotein A and B being similar patients with hypomagnesemia and patients with normal serum Mg [107]. Lee et al. (2015) examined the relationship between low serum Mg and coronary artery calcification in a cross-sectional study which included 34,553 subjects who underwent coronary multi-detector computer tomography and serum Mg measurement as part of a health program in Korea. After differentiating the cohort based on their serum Mg levels, and dividing them into three groups (low < 1.9 mg/dL, normal 1.9–2.3 mg/dL, high > 2.3 mg/dL), the mean values of TC, LDL-C and HDL-C were compared, but no differences in serum lipids were seen based on the Mg subgroups [108].

#### 3.4.4. Hypertension

Zemel et al. (1990) explored the benefits of Mg supplementation during a 3-month RCT, but hypertensive patients displayed no significant changes in serum lipids [109]. On the other hand, Motoyama et al. (1989)’s prescription of 600 mg of Mg oxide for 4 weeks to 21 hypertensive men resulted in significant decreases in TG and free fatty acids [110]. The 4-month supplementation with 2.5 MgCl_2_ (450 mg Mg) employed by Guerrero-Romero and Rodriguez-Moran (2008) in their RCT led to significant improvements in HDL-C levels versus placebo in subjects diagnosed with hypertension [111]. In the prospective, 3-year observational Esfandiari et al. (2017)’s study tracking the effects of the adherence to the DASH diet, it was found that a higher questionnaire-assessed DASH score was associated with a higher Mg consumption and lower TC (*p* < 0.05) [112]. Cunha et al. (2016) performed an RCT to investigate oral Mg supplementation and its effects on the improvement of endothelial function and subclinical atherosclerosis in thiazide-treated hypertensive women. There was a significant difference in HDL-C and LDL-C between the placebo group (n = 18) and the Mg supplemented group (n = 17) at the start of the trial compared to the mean values determined at the end of the clinical trial, but Mg administration failed to alter the HDL-C (*p* = 0.720) or LDL-C (*p* = 0.058) concentrations [113]. As a strategy to combat hypertension, Karppanen et al. (1984) examined the benefits of KCl–NaCl–MgCl_2_ (low in sodium) salts as a replacement for the common table salt (NaCl) used for food preparation. Multiple measurements were made before, after, and during the time that the patients received the salt mixture, including TC and TG. Although serum Mg significantly increased, no statistically significant changes in TC or TG were seen [114]. Delva et al. (1998) discovered that low intra-lymphocytic Mg levels correlated with an increase in TG, regardless of the patients’ hypertensive or normotensive status [115].

The most relevant information of this subsection are summarized in Table 5.

### 3.5. Crosstalk of Magnesium, Chronic Kidney Disease, and Hemodialysis 

A total of 16 studies assessed the crosstalk of Mg, serum lipids, and kidney disorders, focusing on the relationship of serum Mg with the lipid profile in chronic kidney disease (CKD) or in patients undergoing hemo-/peritoneal dialysis. The impact of dietary Mg intake or Mg supplementation on serum lipids was also scrutinized.

#### 3.5.1. Chronic Kidney Disease

Toprak et al. (2017) evaluated the impact of hypomagnesemia on erectile dysfunction in 372 elderly, non-T2DM, stage 3 and 4 CKD patients and argued that the subjects with hypomagnesemia were more likely to suffer from obesity (*p* = 0.003), MetS (*p* = 0.026), have increased waist circumference (*p* = 0.043) and low HDL-C (*p* = 0.009) [116]. Khatami et al. (2013) evaluated the relationship between serum Mg and the lipid profile in 103 patients diagnosed with end-stage renal disease who were receiving hemodialysis. Serum Mg was similar between patients who had a history of dyslipidemia or had received statins, and there were no differences in terms of HDL-C, LDL-C, or apoprotein(a) levels between subjects with low versus high serum Mg concentrations. However, TC (*p* = 0.03) and TG (*p* = 0.04) were elevated in individuals with high serum Mg levels, though no correlations were detected between Mg and TC, TG, LDL-C, HDL-C or apoprotein(a) concentrations [117]. Dey et al. (2015) also evaluated the links between serum Mg concentrations and the lipid profile in 90 patients diagnosed with CKD. CKD subjects had lower serum and urinary Mg and higher TC, LDL-C, and non-HDL-C (*p* < 0.001 for all) versus controls, yet no differences were recorded in terms of HDL-C, TG, or VLDL concentrations. In patients suffering from CKD, there was a positive correlation of serum Mg with HDL-C (r = 0.326, *p* = 0.002) and negative correlations with TC (r = −0.247, *p* = 0.019), LDL-C (r = −0.303, *p* = 0.004), and non-HDL-C (r = −0.289, *p* = 0.006). Serum Mg also correlated with the Framingham risk score (r = −0.939, *p* < 0.001), the presence of MetS (r = −0.830, *p* < 0.001) and CKD severity (r = −0.245, *p* = 0.02) [118]. Cambray et al. (2020) analyzed the Mg-lipids-atherosclerosis crosstalk in 1754 CKD patients, revealing that Mg and TG concentrations displayed a tendency to increase (*p* < 0.001 for trend, both groups) and TC, LDL-C, and HDL-C displayed a tendency to decrease (*p* < 0.001 for trend for all) as CKD severity advanced toward dialysis. Mg levels were correlated with the presence of T2DM (r = −0.070, *p* = 0.003), hypertension (r = 0.053, *p* = 0.028), BMI (r = −0.053, *p* = 0.027), however no associations of Mg with the presence of dyslipidemia or TC, TG, HDL-C, or LDL-c were detected. In the multivariate linear effects model for carotid intima-media thickness, the authors reported associations between the aforementioned marker of atherosclerosis and TC (β = −0.006, SE = 0.003 *p* = 0.02), HDL-C (β = 0.006, SE = 0.003, *p* = 0.03), LDL-C (β = 0.006, SE = 0.003, *p* = 0.04), and TG (β = 0.001, SE = 0.0005, *p* = 0.014), but not with Mg (β = −0.12, SE = 0.099, *p* = 0.23). Despite these findings, when looking closely at the interactions of Mg with serum lipids, namely Mg-TC (β = 0.008, SE = 0.003, *p* = 0.011), Mg-HDL-C (β = −0.007, SE = 0.003, *p* = 0.016), Mg-LDL-C (β = −0.007, SE = 0.003, *p* = 0.03), and Mg-TG (β = −0.0014, SE = 0.0005, *p* = 0.01), Mg concentrations and carotid intima-media thickness were associated [119]. In a pilot study conducted on kidney transplant patients suffering from hypomagnesemia, the administration of Mg oxide significantly decreased TC and LDL-C, but did not alter TC/HDL-C, TG or apolipoprotein fractions [120].

#### 3.5.2. Hemodialysis

Liu et al. (2013) also evaluated the associations between serum Mg and the lipid profile in hemodialysis patients, revealing that subjects suffering from hypo- versus hypermagnesemia had higher HDL-C (*p* < 0.05) but similar LDL-C, TC, TG, and lipoprotein-a concentrations. Among the aforementioned components of the lipid profile, Mg levels only correlated with HDL-C (r = −0.028, *p* = 0.024) [121]. Hemodialysis reduced serum Mg levels (1.11 ± 0.14 mmol/L versus 0.97 ± 0.10 mmol/L, *p* < 0.05) in 148, with Han et al. (2020) reporting that pre-hemodialysis Mg concentrations are correlated with TC [β = 0.03 (0.006, 0.05), *p* = 0.016; β = −0.003 (−0.004, −0.0009), *p* = 0.003; β = 0.03 (0.006, 0.05), *p* = 0.02] according to different un/adjusted models [122]. According to Mortazavi et al. (2013)’s RCT, a 6-month Mg supplementation reduced LDL-C (*p* = 0.04) but did not affect HDL-C, TC, or TG in 54 Iranian subjects undergoing hemodialysis [123]. Shimohata et al. (2019) investigated 83 patients without T2DM who were undergoing hemodialysis in order to assess the link between mortality and serum Mg concentrations and revealed an univariate association between Mg levels and HDL-C (r = 0.284, *p* = 0.009) but not LDL-C (r = 0.075, *p* = 0.499). However, when analyzed by multiple regression, the Mg-HDL-C association did not reach statistical significance (coefficient: 0.004; β = 0.196; *p* = 0.105). Nevertheless, the mortality was higher (log rank = 4.951; *p* = 0.026) in patients with Mg < 2.5 mg/dL versus ≥2.5 mg/dL [124]. Robles et al. (1997) have also shown that in patients receiving hemodialysis there are significant correlations of low serum Mg levels with increased LDL-C, VLDL-C, and apoB levels (*p* < 0.001) [125]. Tamura et al. (2019) scrutinized 392 patients undergoing hemodialysis for 4 years and concluded that low Mg levels are associated with higher mortality rates. In addition, in their paper, there were positive Mg–TC (*p* = 0.257), and Mg–TG (*p* = 0.0279) associations. However, HDL-C remained unchanged regardless of the Mg concentrations (*p* = 0.097) [126]. Ansari et al. (2012) observed significant positive correlations between serum Mg and lipoprotein-a (r = 0.40, *p* < 0.007), HDL-C (r = 0.31, *p* < 0.01) and TG (r = 0.35, *p* < 0.005), but not with LDL-C or TC in 50 patients receiving hemodialysis [127]. Similarly, Baradaran and Nasri (2004) studied 36 hemodialysis subjects and unmasked significant positive correlations of serum Mg with lipoprotein (a) (r = 0.65, *p* < 0.05) and TG (r = 0.32, *p* < 0.05), but not with TC, HDL-C, or LDL-C [128]. HDL-C (r = 0.315, *p* = 0.003) was positively associated with Mg concentrations in Ikee et al. (2016)’s cross-sectional study which included eighty-six patients undergoing hemodialysis [129]. Mitwalli et al. (2016) studied the significance of lower Mg levels in the serum of Saudi dialysis patients. One hundred and fifteen patients partook in this retrospective study: seventy patients were on hemodialysis and forty-five were on peritoneal dialysis. The subjects’ serum values of Mg, TC, and TG were determined, showing that patients that underwent peritoneal dialysis had lower Mg levels compared to hemodialysis patients. The correlation studies did not find statistically significant associations between Mg and TC or TG variations [130]. While studying the association of hypomagnesemia with increased mortality among patients that underwent peritoneal dialysis in a research which included two hundred and fifty three subjects, Cai et al. (2016) reported a positive association between low serum Mg and TG (r = 0.160, *p* = 0.011), but the correlation with TC did not reach statistical significance (*p* = 0.929) [131].

The most relevant information of this subsection are summarized in Table 6.

Finally, the effects of Mg supplementation (alone, in combination or as part of dietary interventions) as depicted in RCTs are reported in Table 7.

## 4. Discussion

In this systematic review, we focused on depicting the relationship between Mg levels and serum lipids in dyslipidemia and associated disorders. We analyzed a total of 124 studies conducted on patients diagnosed with dyslipidemia, MetS, T2DM, cardiovascular or kidney disorders, yet due to the heterogeneity of the analyzed papers it is difficult to conclude to which extent Mg levels are linked to serum lipids concentrations. Although we aimed to investigate both “classical” (TC, TG, HDL-C and LDL-C) and “non-classical” components (apolipoproteins, Lp(a), oxLDL etc.) of the lipid profile, most studies were focused on examining the crosstalk of Mg levels and TC, TG, HDL-C, and LDL-C.

Mg deficits may arise both from primary (insufficient intake, decreased absorption or elevated excretion) and secondary causes, e.g., disorders that accompany the advancement in age, several comorbidities (T2DM, MetS) or it can occur due to the use of certain medications such as loop diuretics [132]. In addition, particular attention should be given to the methods employed in the measurement of Mg concentrations. Barbagallo et al. (2014) demonstrated that, in elderly patients diagnosed with T2DM, serum ionized rather than total serum Mg may emerge as a superior predictor of the subclinical deficit of this micronutrient. In addition, they also detected that TG may be a confounding factor in the crosstalk between Mg levels and markers of glucose metabolism. For example, after multiple adjustments for TG, BMI, and glomerular filtration rate, the associations of serum total Mg with FPG and HbA1c, respectively, failed to reach statistical significance. However, serum-ionized Mg remained associated with these variables despite multiple adjustments [133]. Moreover, in their recent umbrella review of systematic reviews and meta-analyses of observational and intervention studies focused on the crosstalk between Mg concentrations and health outcomes, Veronese et al. (2019) evidenced that an elevated intake of this micronutrient can result in a reduction of the risk of both stroke and T2DM. However, Mg intake was not linked to any other cardiovascular endpoints based on their results [134].

The potential lipid-lowering effects of Mg warrant further investigation, with a myriad of studies linking the serum concentrations of this micronutrient to cardiometabolic disorders, e.g., obesity, T2DM, MetS, cardiovascular disorders, neurological ailments, and even cancer, all of which are worldwide public health threats [5,135,136]. Mg supplements stand out as one of the most popular supplements in Europe and the United States [137]. In particular, Mg orotate supplementation, due to the Mg-fixing capacity of this salt, has exerted health benefits [137,138]. For example, in an RCT, patients with heart failure who were prescribed Mg orotate had better 1-year survival versus subjects receiving placebo [138]. Similarly, patients diagnosed with concomitant heart failure and hypertension who were administered Mg orotate registered a decrease in both blood pressure and N-terminal (NT)-pro hormone BNP (NT-proBNP) [139].

In terms of Mg supplementation, the most reliable data included in our paper were obtained from 29 RCTs with a total number of 1724 subjects who received different forms of Mg supplementation for a period of time ranging from 4 to 24 weeks [13,14,18,35,36,38,41,42,43,53,80,82,83,87,88,89,90,91,92,93,95,96,98,99,105,109,111,113,123]. Overall, the vast majority of the analyzed RCTs reported no variations in HDL-C (n = 19), LDL-C (n = 16), TG (n = 17), TC (n = 14), TC/HDL-C (n = 4), VLDL (n = 2), or LDL-C/HDL-C (n = 1). However, some RCTs reported elevations of the HDL-C (n = 8), apoA1 (n = 1), or HDL-C/TG (n = 1), as well as reductions in LDL-C (n = 7), TC (n = 4), TG (n = 7), TC/HDL-C (n = 2), or VLDL (n = 3) in the participants exposed to the Mg intervention [13,14,18,35,36,38,41,42,43,53,80,82,83,87,88,89,90,91,92,93,95,96,98,99,105,109,111,113,123].

Our systematic review has several strengths and limitations. A major strength of our manuscripts is that we systematically analyzed a considerable number of studies that focused on the assessment of the relationship between Mg levels and serum lipids concentrations. Although lengthy, we strived to summarize all the available evidence regarding this research question and clarify the crosstalk between Mg and serum lipids in dyslipidemia and associated disorders. We also reported data derived from RCTs conducted with the purpose of evaluating the impact of Mg supplementation on serum lipids values. However, due to multiple reasons (the data analyzed was extremely heterogeneous, most studies included small sample sizes and no control group, some studies assessed serum Mg whereas other assessed hair or urinary Mg concentrations, the methods employed to measure Mg levels were heterogeneous, the crosstalk between Mg and serum lipids was evaluated in a myriad of disorders), we did not perform a meta-analysis of the gathered evidence. Thus, further research is needed to clarify the relationship between Mg and serum lipids levels, as well as the effects of Mg supplementation on the lipid metabolism. 

## 5. Conclusions

Mg remains an important micronutrient for human health, and its putative role in the pathogenesis of dyslipidemia and associated disorders, i.e., MetS, T2DM and cardiovascular disease, has been documented in a myriad of studies. However, the mechanisms activated by Mg in its interplay with serum lipids have been elucidated, and further research is warranted to explore the lipid-lowering effects of Mg supplementation or whether a higher dietary intake of this element might emerge as a spearhead in the therapeutic *armamentarium* of dyslipidemia and closely linked diseases. 

## Figures and Tables

**Table 1:** Magnesium and serum lipids interplay in patients with a normal health status and patients diagnosed with dyslipidemia or related disorders.

Author and Year	Condition	Number of Patients	Method of Mg Determination	Main Results
Barragán et al. (2020) [8]	Cardiometabolic risk factors: T2DM, hypercholesterolemia, hypertension	492	Serum, urine (spectrometry)	Prevalence of hypoMg = 19% Hypercholesterolemia: Mg ↑; T2DM: Mg ↓ HypoMg-T2DM association (OR: 3.36, 95% CI: 1.26–8.96, *p* = 0.016) TC, LDL-C associated with Mg quartiles ↑ Hypercholesterolemia in 4th versus 1st quartile of Mg levels (OR: 3.12; 95% CI: 1.66–5.85, *p* < 0.001)
Bersohn and Oelofse (1957) [9]	Healthy	100	Serum (spectrophotometry)	Normal TC—normal Mg ↓ TC–↑ Mg Hypercholesterolemia: ↓ Mg
Petersen et al. (1976) [10]	Hypercholesterolemia Hypertriglyceridemia	17	Serum (spectrophotometry)	3 g/day of MgO for 6 weeks: no effect on TC, TG
Liu et al. (2020) [11]	Dyslipidemia	1466	Serum (spectrophotometry)	↑ TC, TG, HDL-C and LDL-C (*p* < 0.001) ↓ Mg (*p* = 0.002) ↑ Dyslipidemia in the 4th versus 1st Mg quartile (OR = 1.4, 95% CI 1.0–1.9, *p* = 0.023)
Jin and Nicodemus-Johnson (2018) [12]	Healthy	12,284	Serum (method unspecified)	(+) association of Mg intake and HDL-C in ♀ (−) association of Mg intake and TC/HDL-C ratio in ♀ (−) association of Mg intake and TG in ♀ and ♂
Randell et al. (2008) [15]	Healthy	1318	Serum (spectroscopy)	(+) association of Mg and TC, HDL-C, LDL-C, TG
Guerrero-Romero et al. (2019) [16]	Healthy	529	Serum (method unspecified)	Mg and K supplementation ↓ TG
Fu et al. (2012) [19]	Healthy	42	Serum (method unspecified)	deep sea water (395 Mg mg/L) ↓ TC, LDL-C and HDL-C versus MgCl_2_ fortified (386 Mg mg/L) or reverse osmotic water
Nerbrand et al. (2003) [20]	CV risk factors	207	Serum, whole blood, muscle, urine (method unspecified)	No correlation of Mg content in water and CV risk factors
Luoma et al. (1973) [21]	CV risk factors	300	Serum (method unspecified)	No correlation of Mg in drinking water and CV risk factors (TG, TC)
Balliett et al. (2013) [22]	Healthy	49	Serum (method unspecified)	Low-energy dietary interventions (+ Mg) ↓ TC, LDL-C, TG and HDL-C
de Los Rios (1963) [23]	Schizophrenia	28	Serum (method unspecified)	Controlled diet (Mg > RDV): no associations of ΔTC and ΔMg
Cocate et al. (2013) [24]	Healthy	296	Serum (method unspecified)	Association of ↑Mg intake and oxLDL
Ruel et al. (2014) [25]	Multimorbidity, Hypercholesterolemia	1020	Serum (method unspecified)	↑ consumption of cereals (r = 0.60, *p* < 0.0001), fruits and vegetables (r = 0.49, *p* < 0.0001) associated with ↑ Mg and ↓ multimorbidity
Bain et al. (2015) [26]	Healthy	4443	Serum (method unspecified)	Inverse correlation of Mg intake and TC in ♂ (*p* = 0.02) and ♀ (*p* = 0.04)
Samavarchi Tehrani et al. (2020) [27]	Dyslipidemia	447	Serum (method unspecified)	Mg levels associated with dyslipidemia in embolic stroke (*p* = 0.012)
Kim et al. (2014) [28]	Healthy	1566	Serum (method unspecified)	(+) association of Mg intake and HDL-C (*p* = 0.001), HDL-2 (*p* = 0.018), HDL-3 (*p* = 0.013)
Kim and Choi (2013) [29]	Healthy	258	Serum (method unspecified)	↑ Mg intake, ↑ TG (*p* < 0.05), ↓ HDL-C (*p* < 0.01), ↑ AIP (*p* < 0.001) in ♂
Cao et al. (2015) [30]	Healthy	2837	Serum, urine (method unspecified)	(+) association of ↑ serum Mg and TC (*p* < 0.001), HDL-C (*p* < 0.001), LDL-C (*p* = 0.001), TG (*p* < 0.001) (−) association of ↑ serum Mg and non-HDL-C/HDL-C (*p* = 0.003) (+) association of Mg/creatinine ratio in urine and TC (*p* = 0.004), HDL-C (*p* = 0.003) and LDL-C (*p* = 0.009)
López-González et al. (2014) [31]	Post-menopause	78	Serum (method unspecified)	Mg in red blood cells, not serum Mg, correlates with TG (r = 0.287, *p* = 0.011)
Yamori et al. [32]	Hypercholesterolemia	4211	Serum, urine (method unspecified)	↑ hypercholesterolemia, ↑ TC in lower versus higher 24-h urinary Mg/creatinine ratios (*p* < 0.001 for trend for both) ↑ hypercholesterolemia in the lowest versus the highest quintiles of 24-h Mg/creatinine urinary ratios (OR = 2.73; 95% CI 2.03 to 3.67; *p* < 0.001)
Guerrero-Romero and Rodriguez-Moran (2013) [33]	Overweight/obesity	427	Serum (method unspecified)	NW and MHO: ↑ Mg (*p* = 0.04 and *p* = 0.01, respectively), ↓ TG (*p* < 0.0005 for both) MONW: (−) Mg-TG correlation Obese and non-obese: ↓ Mg associated with hypertriglyceridemia (OR = 1.61, 95% CI: 1.5–2.46 and OR = 6.67, 95% CI 2.1–20.4, respectively)
Lefebvre et al. (2014) [34]	Obesity	267	Serum (method unspecified)	No Mg-TG correlation in candidates for bariatric surgery
Guerrero-Romero et al. (2016) [37]	Obesity	681	Serum (method unspecified)	↓ HDL-C, ↑ TG in ↓ Mg versus normal Mg groups
Farsinejad-Marj et al. (2020) [39]	PCOS	60	Serum (method unspecified)	250 mg/day Mg oxide for 8 weeks: no effect on TC, HDL-C, LDL-C, TG, TC/HDL-C, TG/HDL-C
Cutler et al. (2019) [40]	PCOS	137	Serum (method unspecified)	Mg-rich diet ↓ insulin resistance and ↑ HDL-C (*p* = 0.02 for both)

Mg, magnesium. T2DM, type 2 diabetes mellitus. CV, cardiovascular. PCOS, polycystic ovary syndrome. hypoMg, hypomagnesemia. OR, odds ratio. CI, confidence interval. ↑, increased. ↓, decreased. (+), positive. (−), negative. ♂, male. ♀, female. MgO, Mg oxide. HDL-C, high-density lipoprotein cholesterol. LDL-C, low-density lipoprotein cholesterol. TC, total cholesterol. TG, triglycerides. VLDL, very low-density lipoprotein cholesterol. apoA1, apolipoprotein A1. oxLDL, oxidized LDL. RDV, recommended dietary value. Δ, variation. MgCl_2_, Mg chloride. mg, milligrams. g, grams. mL, milliliter. K, potassium. MONW, metabolically obese normal-weight. NW, normal weight.

**Table 2:** Magnesium and serum lipids interplay in MetS.

Author and Year	Number of Patients	Method of Mg Determination	Main Results
Ali et al. (2013) [44]	213	Unspecified	90% ♂ Mg intake < daily recommended dose (↓ whole grains, vegetable intake)
Vajdi et al. (2020) [45]	588	Unspecified	↓ odds of MetS (*p* = 0.01), ↓ LDL-C (*p* = 0.04), in the 1st versus 4th quartile of plant source-based diets (↑ Mg)
Akbarzade et al. (2020) [46]	850	Unspecified	No association of nutrient patterns with ↑ Mg intake and MetS components
Mottaghian et al. (2020) [47]	1637	Unspecified	No association of Mg-rich dietary pattern and lipid profile changes ↑ TG (*p* < 0.05 for trend) across the quartiles of the Mg-rich nutrient pattern
Choi and Bae (2013) [48]	5136	Unspecified	♀ and ♂: ↓ Mg intake, no link with MetS ♀ with ↓ HDL-C: ↓ Mg intake ♀ and ♂ with ↑ TG: no link with Mg intake
Cano-Ibáñez et al. (2019) [49]	6646	Unspecified	MetS: ↓ Mg intake ↓ Mg intake in ♂ and ↓ education
Choi et al. (2014) [50]	456	Serum (automatic analytical analyzer)	serum Mg similar in MetS versus controls ↓ hair Mg levels (*p* = 0.046) ↑ Na/Mg ratio (*p* = 0.013) in MetS (−) correlation of hair Mg and TG (r = −0.125, *p* < 0.05)
Vanaelst et al. (2012) [51]	166	Unspecified	(−) correlations of serum Mg and non-HDL-C (r = −0.170, *p* = 0.030), metabolic score (r = −0.257, *p* = 0.001) ↓ hair Mg (*p* = 0.015) in ♀ with metabolic score > 3 points
Sun et al. (2013) [52]	7641	Serum (biochemical analyzer)	↓ Mg (*p* < 0.001), ↓ HDL-C, ↑ TC, ↑ TG, ↑ LDL-C
Rotter et al. (2015) [54]	313	Serum and whole blood (spectrometry)	↓ Mg in MetS (*p* = 0.02), T2DM (*p* = 0.0001), HTN (*p* = 0.0001) (+) associations of Mg and TC (r = 0.25; *p* < 0.001), LDL-C (r = 0.26; *p* < 0.001)
Ghasemi et al. (2010) [55]	137	Serum (spectrometry)	↓ Mg in patients > 60 years with MetS, T2DM and hyperglycemia
Evangelopoulos et al. (2008) [56]	117	Serum (colorimetric reaction)	(+) Mg-HDL-C association (r = 0.18; *p* = 0.05)
Guerrero-Romero and Rodríguez-Morán (2002) [57]	576	Serum (colorimetric assay)	(+) Mg-HDL-C association (r = 0.36, *p* < 0.05) (−) Mg-TC association (r = −0.29, *p* < 0.05)
Yuan et al. (2016) [58]	408	Serum (spectrometry)	(+) correlations: Mg-BMI (r = 0.128, *p* < 0.05), Mg-TC (r = 0.254, *p* < 0.05), Mg-LDL-C (r = 0.280, *p* < 0.05)
Rotter et al. (2016) [59]	313	Serum (spectrometry)	(+) correlations: Mg-TC (r = 0.25, *p* < 0.0001), Mg-LDL-C (r = 0.26, *p* < 0.0001)

Mg, magnesium. MetS, metabolic syndrome. T2DM, type 2 diabetes mellitus. HTN, hypertension. hypoMg, hypomagnesemia. OR, odds ratio. CI, confidence interval. ↑, increased. ↓, decreased. (+), positive. (−), negative. ♂, male. ♀, female. MgO, Mg oxide. HDL-C, high-density lipoprotein cholesterol. LDL-C, low-density lipoprotein cholesterol. TC, total cholesterol. TG, triglycerides. VLDL, very low-density lipoprotein cholesterol. apoA1, apolipoprotein A1. oxLDL, oxidized LDL. RDV, recommended dietary value. Δ, variation. MgCl_2_, Mg chloride. mg, milligrams. g, grams. mL, milliliter. K, potassium. MONW, metabolically obese normal-weight. NW, normal weight.

**Table 3:** Magnesium and serum lipids interplay in T2DM.

Author and Year	Number of Patients	Method of Mg Determination	Main Results
van Dijk et al. (2019) [60]	5568	Serum (xylidyl blue test)	(−) correlation of Mg (measured by nuclear magnetic resonance spectroscopy and colorimetric assays) and TG (r = −0.184, *p* = 0.002 and r = −0.194, *p* = 0.001, respectively)
Rusu et al. (2013) [61]	154	Serum (automated multianalyzer)	T2DM + peripheral arterial disease: ↓ Mg (*p* < 0.01), ↓ HDL-C, ↑ TG, ↑ TC
Spiga et al. (2019) [62]	589	Serum (colorimetric assay)	↓ Mg, ↓ HDL-C, ↑ TG in normal glucose tolerance → impaired fasting glucose → T2DM (*p* < 0.02, *p* < 0.01 and *p* < 0.001, respectively) (+) correlations: Mg and TC (r = 0.154, *p* < 0.001), HDL-C (r = 0.113, *p* < 0.01), LDL-C (r = 0.170, *p* < 0.001) ↑ Mg: ↓ risk of T2DM (OR = 0.765, 95% CI 0.629–0.932, *p* < 0.01)
Esmeralda et al. (2021) [63]	62	Serum and urine analysis (methods unspecified)	T2DM: ↑ TG (*p* = 0.004), ↑ fractional excretion of Mg (*p* = 0.01), ↓ serum Mg (*p* = 0.001) versus controls
Gopal et al. (2019) [64]	90	Serum (calmagite colorimetric test)	Mg concentrations predict proliferative retinopathy development in T2DM (optimum cut-off 1.7 mg/dL, sensitivity 92.86%, specificity 77.14%, AUC 0.837, SEM 0.06, 95% CI 0.70–0.92) Mg ↓: no retinopathy → non-proliferative retinopathy → proliferative retinopathy (*p* < 0.01) proliferative retinopathy: ↑ HDL-C (*p* < 0.05), similar TC, TG, LDL-C, VLDL
Hruby et al. (2017) [65]	202,743	Unspecified	15% T2DM-risk ↓ in individuals with ↑ dietary intake of Mg ↑ Hypercholesterolemia in the 5th (12.7% for 427–498 mg/day) versus 1st first (8.7% for 242–275 mg/day) quartile of Mg intake
Anetor et al. (2002) [66]	60	Serum (spectrophotometry)	(+) association of Mg and TC (r = 0.6; *p* < 0.001)
Corica et al. (2006) [67]	290	Serum (ion selective analyzer)	↓ Mg: ↓ HDL-C (*p* < 0.001) and ↑ TG (*p* < 0.001)
Romero and Moran (2000) [68]	390	Serum (chemical autoanalyzer)	↓ Mg associated with ↓ HDL-C (*p* = 0.01 in T2DM; *p* = 0.05 in IFG; *p* = 0.03 for controls)
Yu et al. (2018) [69]	8163	Serum (xylidyl blue test)	↑ serum lipids, except for HDL-C, across progressive Mg concentrations (from ↓ Mg of ≤0.65 mmol/L, normal 0.65–0.95 mmol/L, ↑ levels ≥0.95 mmol/L) (*p* < 0.05) ↑ TG, TC, HDL-C and LDL-C (Mg ≥0.95 mmol/L versus ↓ Mg, *p* < 0.05)
Kurstjens et al. (2016) [70]	395	Serum (spectrophotometry)	(−) negative correlation of Mg and TG (r = −0.273, *p* < 0.001) (+) positive correlation of Mg and HDL-C (r = 0.156, *p* = 0.002)

Mg, magnesium. MetS, metabolic syndrome. T2DM, type 2 diabetes mellitus. HTN, hypertension. hypoMg, hypomagnesemia. OR, odds ratio. CI, confidence interval. ↑, increased. ↓, decreased. (+), positive. (−), negative. ♂, male. ♀, female. MgO, Mg oxide. HDL-C, high-density lipoprotein cholesterol. LDL-C, low-density lipoprotein cholesterol. TC, total cholesterol. TG, triglycerides. VLDL, very low-density lipoprotein cholesterol. apoA1, apolipoprotein A1. oxLDL, oxidized LDL. RDV, recommended dietary value. Δ, variation. MgCl_2_, Mg chloride. mg, milligrams. g, grams. mL, milliliter. K, potassium. MONW, metabolically obese normal-weight. NW, normal weight.

**Table 4:** Interplay of hypomagnesemia and T2DM.

Author and Year	Number of Patients	Method of Mg Determination	Main Results
Rasheed et al. (2012) [71]	319	Serum (spectrophotometry)	↑ prevalence of hypoMg in T2DM hypoMg: ↑ TG, ↑ LDL-C, ↓ HDL-C
Srinivasan el al. (2012) [72]	30	Serum (calmagite colorimetric test)	association of hypoMg and TG (*p* < 0.05)
Pokharel et al. (2017) [73]	300	Serum (xylidyl blue test)	↑ prevalence of hypoMg in T2DM: 50% (−) correlation of Mg and TC (r = −0.219; *p* < 0.01), LDL-C (r = −0.168; *p* < 0.05)
Hyassat et al. (2014) [74]	1105	Serum (colorimetric assay)	↑ prevalence of hypoMg in overweight/obese T2DM: 19% dyslipidemia, statin use associated with hypoMg (*p* = 0.022 and *p* < 0.001, respectively) hypoMg-statin use association (OR = 1.56, 95% CI: 1.1–2.2)
Waanders et al. (202p) [75]	929	Serum (colorimetric assay)	(+) associations of Mg and TC (r = 0.142, *p* < 0.001), TC/HDL-C ratio (r = 0.114, *p* < 0.001), LDL-C (r = 0.166, *p* < 0.001) stepwise multivariable regression: Mg associated with LDL-C (β = 0.141, *p* = 0.001)
Huang et al. (2012) [76]	210	Serum (methylthymol blue method)	T2DM, >65 years: insufficient Mg intake (>88%), hypoMg (37%) (+) association of Mg intake and HDL-C (r = 0.192; *p* = 0.005)
Corsonello et al. (2000) [77]	110	Serum (ion selective analyzer)	↓ Mg, ↑ TG with the ↑ urinary protein loss (*p* < 0.001) hypoMg: ↑ TC
Shardha et al. (2014) [78]	358	Serum (method unspecified)	↑ TG (*p* = 0.001), ↑ LDL-C (*p* < 0.001), ↓ HDL-C (*p* < 0.001) in T2DM + hypoK + hypoMg

Mg, magnesium. MetS, metabolic syndrome. T2DM, type 2 diabetes mellitus. HTN, hypertension. hypoMg, hypomagnesemia. OR, odds ratio. CI, confidence interval. ↑, increased. ↓, decreased. (+), positive. (−), negative. ♂, male. ♀, female. MgO, Mg oxide. HDL-C, high-density lipoprotein cholesterol. LDL-C, low-density lipoprotein cholesterol. TC, total cholesterol. TG, triglycerides. VLDL, very low-density lipoprotein cholesterol. apoA1, apolipoprotein A1. oxLDL, oxidized LDL. RDV, recommended dietary value. Δ, variation. MgCl_2_, Mg chloride. mg, milligrams. g, grams. mL, milliliter. K, potassium. MONW, metabolically obese normal-weight. NW, normal weight. hypoK, hypokalemia.

**Table 5:** Interplay of magnesium and serum lipids in cardiovascular disorders.

Author and Year	Condition	Number of Patients	Method of Mg Determination	Main Results
Qazmooz et al. (2020) [101]	atherosclerosis versus unstable angina	178	Serum (spectrophotometry)	↑ Mg in atherosclerosis versus controls and unstable angina Mg explained the variance in AIP (β = −0.205, t = −3.036, *p* = 0.003), Castelli index 1 (β = −0.179, t = −2.633, *p* = 0.009), Castelli index 2 (β = −0.143, t = −1.983, *p* = 0.049), HDL-C (β = 0.157, t = 2.106, *p* = 0.037)
Brown et al. (1958) [102]	myocardial infarction	1225	Serum (spectrophotometry)	Mg not associated with serum cholesterol, total lipids, α/β lipoproteins
Mahalle el al. (2012) [103]	cardiovascular disease	300	Serum (xylidyl blue test)	↓ Mg: ↑ TC, ↑ LDL-C, ↑ VLDL, ↑ TG and ↓ HDL-C
Liao et al. (1998) [104]	CHD	13,922	Serum (calmagite colorimetric test)	↑ Mg = ↑ TC, ↑ HDL-C, ↓ LDL-C
Petersen et al. (1977) [106]	CHD	73	Serum, erythrocytes (spectrophotometry)	(+) correlation of erythrocyte Mg and TC (r = 0.25, *p* < 0.05)
Posadas-Sanchez et al. (2016) [107]	CAC	1276	Serum (xylidyl blue test)	hypoMg and normoMg: similar LDL-C, HDL-C, TG, apolipoprotein A/B
Lee et al. (2015) [108]	CAC	34,553	Serum (colorimetric assay)	no TC, LDL-C, HDL-C difference across Mg subgroups: ↓ < 1.9 mg/dL, normal 1.9–2.3 mg/dL, ↑ > 2.3 mg/dL
Esfandiari et al. (2017) [112]	HTN	927	Unspecified	↑ questionnaire-assessed DASH score = ↑ Mg and ↓ TC (*p* < 0.05)
Karppanen et al. (1984) [114]	HTN	126	Serum (method unspecified)	KCl–NaCl–MgCl_2_ salts versus common table salt (NaCl) ↑ Mg but no effect on TC, TG
Delva et al. (1998) [115]	HTN	52	Intralymphocyte (fluorimetric test)	↓ intra-lymphocytic Mg associated with ↑ TG

Mg, magnesium. MetS, metabolic syndrome. T2DM, type 2 diabetes mellitus. HTN, hypertension. hypoMg, hypomagnesemia. OR, odds ratio. CI, confidence interval. ↑, increased. ↓, decreased. (+), positive. (−), negative. MgO, Mg oxide. HDL-C, high-density lipoprotein cholesterol. LDL-C, low-density lipoprotein cholesterol. TC, total cholesterol. TG, triglycerides. VLDL, very low-density lipoprotein cholesterol. apoA1, apolipoprotein A1. oxLDL, oxidized LDL. RDV, recommended dietary value. Δ, variation. MgCl_2_, Mg chloride. mg, milligrams. g, grams. mL, milliliter. K, potassium. MONW, metabolically obese normal-weight. NW, normal weight.

**Table 6:** Interplay of magnesium, serum lipids, and the kidney.

Author and Year	Condition	Number of Patients	Method of Mg Determination	Main Results
Toprak et al. (2017) [116]	stage 3 and 4 CKD, erectile dysfunction	372	Unspecified	hypoMg: ↑ obesity (*p* = 0.003), ↑ MetS (*p* = 0.026), ↓ HDL-C (*p* = 0.009)
Khatami et al. (2013) [117]	end-stage renal disease + HD	103	Serum (spectrophotometry)	↑ Mg = ↑ TC (*p* = 0.03), ↑ TG (*p* = 0.04)
Dey et al. (2015) [118]	CKD	180	Serum (chemical autoanalyzer)	↓ serum and urinary Mg ↑ TC, LDL-C, non-HDL-C (*p* < 0.001 for all) (+) correlation of serum Mg and HDL-C (r = 0.326, *p* = 0.002) (−) correlations of serum Mg and TC (r = −0.247, *p* = 0.019), LDL-C (r = −0.303, *p* = 0.004), non-HDL-C (r = −0.289, *p* = 0.006), Framingham risk score (r = −0.939, *p* < 0.001), the presence of MetS (r = −0.830, *p* < 0.001), CKD severity (r = −0.245, *p* = 0.02)
Cambray et al. (2020) [119]	CKD	1754	Serum (Mg reagent)	↑ CKD severity = ↑ Mg and ↑ TG (*p* < 0.001 for trend) ↑ CKD severity = ↓ TC, LDL-C, HDL-C (*p* < 0.001 for) associations of Mg with T2DM (r = −0.070, *p* = 0.003), HTN (r = 0.053, *p* = 0.028), BMI (r = −0.053, *p* = 0.027) association of CIMT and Mg-TC (β = 0.008, SE = 0.003, *p* = 0.011), Mg-HDL-C (β = −0.007, SE = 0.003, *p* = 0.016), Mg-LDL-C (β = −0.007, SE = 0.003, *p* = 0.03), Mg-TG (β = −0.0014, SE = 0.0005, *p* = 0.01) interactions
Gupta et al. (1999) [120]	kidney transplant	14	Serum (method unspecified)	MgO in hypoMg: ↓ TC, ↓ LDL-C
Liu et al. (2013) [121]	HD	98	Serum (colorimetric assay)	↑ HDL-C (*p* < 0.05) in hypo- versus hyperMg similar LDL-C, TC, TG, lipoprotein-a (−) Mg-HDL-C correlation (r = −0.028, *p* = 0.024)
Han et al. (2020) [122]	HD	148	Serum (toluidine blue assay)	HD ↓ Mg (*p* < 0.05) pre-HD Mg correlated with TC [β = 0.03 (0.006, 0.05), *p* = 0.016; β = −0.003 (−0.004, −0.0009), *p* = 0.003; β = 0.03 (0.006, 0.05), *p* = 0.02]
Shimohata et al. (2019) [124]	HD	83	Serum (chemical autoanalyzer)	Mg-HDL-C association (r = 0.284, *p* = 0.009) multiple regression: no Mg-HDL-C (coefficient: 0.004; β = 0.196; *p* = 0.105) ↑ mortality for Mg < 2.5 versus ≥2.5 mg/dL (log rank = 4.951; *p* = 0.026)
Robles et al. (1997) [125]	HD	25	Serum (method unspecified)	↓ Mg correlated with ↑ LDL-C, ↑ VLDL-C, ↑ apoB (*p* < 0.001)
Tamura et al. (2019) [126]	HD	392	Serum (method unspecified)	↓ Mg associated with ↑ mortality (+) Mg–TC (*p* = 0.257) and Mg–TG (*p* = 0.0279) associations
Ansari et al. (2012) [127]	HD	50	Serum (standard method)	(+) correlations of Mg and lipoprotein-a (r = 0.40, *p* < 0.007), HDL-C (r = 0.31, *p* < 0.01), TG (r = 0.35, *p* < 0.005)
Baradaran and Nasri (2004) [128]	HD	36	Dialysis fluid (method unspecified)	(+) correlations of Mg and lipoprotein (a) (r = 0.65, *p* < 0.05), TG (r = 0.32, *p* < 0.05)
Ikee et al. (2016) [129]	HD	86	Serum (xylidyl blue test)	(+) association of Mg and. HDL-C (r = 0.315, *p* = 0.003)
Mitwalli et al. (2016) [130]	HD, PD	115	Dialysis fluid (Mg reagent)	Mg in PD compared to HD no associations of Mg and TC or TG variations
Cai et al. (2016) [131]	PD	253	Dialysis fluid (chemical autoanalyzer)	(+) association of hypoMg and TG (r = 0.160, *p* = 0.011)

Mg, magnesium. MetS, metabolic syndrome. T2DM, type 2 diabetes mellitus. HTN, hypertension. hypoMg, hypomagnesemia. OR, odds ratio. CI, confidence interval. ↑, increased. ↓, decreased. (+), positive. (−), negative. ♂, male. ♀, female. MgO, Mg oxide. HDL-C, high-density lipoprotein cholesterol. LDL-C, low-density lipoprotein cholesterol. TC, total cholesterol. TG, triglycerides. VLDL, very low-density lipoprotein cholesterol. apoA1, apolipoprotein A1. oxLDL, oxidized LDL. RDV, recommended dietary value. Δ, variation. MgCl_2_, Mg chloride. mg, milligrams. g, grams. mL, milliliter. K, potassium. MONW, metabolically obese normal-weight. NW, normal weight. CKD, chronic kidney disease. HD, hemodialysis. PD, peritoneal dialysis. CIMT, carotid intima-media thickness.

**Table 7:** Effects of Mg supplementation in RCTs.

Authors and Year	Country	Intervention	Duration	Condition	No. Subjects	Effects on the Lipid Profile
Itoh et al. (1997) [13]	Japan	Mg(OH)_2_ ~411–548 mg Mg/day	4 weeks	Healthy	33	HDL-C, apoA1: ↑ LDL-C, TC/HDL-C: ↓
Marken et al. (1989) [14]	USA	800 mg/day MgO	60 days	Healthy	50	TC, HDL-C, LDL-C, VLDL, TG: no effect
Aslanabadi et al. (2014) [18]	Iran	1L/day Mg-rich miner water	1 month	Dyslipidemia	69	TC, HDL-C, LDL-C, TG: no effect
Rodriguez-Moran and Guerrero-Romero (2014) [35]	Mexico	30 mL/day MgCl_2_ 5% solution ~382 mg Mg	4 months	MONW + Hypomagnesemia	47	HDL-C: ↑ TG: ↓
Joris et al. (2017) [36]	The Netherlands	350 mg/day Mg	24 weeks	Overweight/Obesity	52	TC, HDL-C, LDL-C, TG, non-esterified fatty acids: no effect
Solati et al. (2019) [38]	Iran	herbal supplement ~300 mg/day MgSO_4_	6 months	Overweight	70	HDL-C, HDL-C/TG: ↑ LDL-C, TG: ↓
Jamilian et al. (2019) [41]	Iran	250 mg/day Mg + 400 mg/day vitamin E	12 weeks	PCOS	60	TG, VLDL: ↓ HDL-C, LDL-C, TC/HDL-C: no effect
Jamilian et al. (2017) [42]	Iran	200 mg/day Mg + 800 mg/day Ca + 8 mg/day Zn + 400 IU vitamin D	12 weeks	PCOS	60	TC, TG, VLDL: ↓ HDL-C, LDL-C: no effect
Karandish et al. (2013) [43]	Iran	350 mg/day Mg + low-calorie diet + physical exercise	90 days	NAFLD	68	TC, HDL-C, LDL-C, TG: no effect
de Lourdes Lima de Souza e Silva (2014) [53]	Brazil	400 mg/day Mg chelate	12 weeks	MetS	72	TC, HDL-C, LDL-C, TG: no effect
Hamedifard et al. (2020) [80]	Iran	250 mg/day MgO + 150 mg/day ZnSO_4_	12 weeks	T2DM + CHD	60	HDL-C: ↑ TC, TC/HDL-C, LDL-C, VLDL, TG: no effect
Rashvand et al. (2019) [82]	Iran	500 mg/day MgO + 1000 mg/day choline bitartrate	2 months	T2DM	96	HDL-C: ↑ TG: ↓ TC, LDL-C/HDL-C, LDL-C: no effect
Whitfield et al. (2016) [83]	New Zealand	53.5 g Mg, Cr, cinnamon enriched honey	40 days	T2DM	12	TC, LDL-C: ↓ HDL-C, TG: no effect
Ham and Shon (2020) [87]	Korea	440 mL/day Mg-enriched deep sea water	8 weeks	Prediabetes	74	TC, LDL-C: ↓ HDL-C, TG: no effect
Cosaro et al. (2014) [88]	Italy	16.2 mmol/day Mg pidolate	8 weeks	Healthy men with a positive family history for T2DM/MetS	14	TC, HDL-C, LDL-C, TG: no effect
Asemi et al. (2015) [89]	Iran	250 mg/day MgO	6 weeks	Gestational diabetes + Mg deficiency	70	HDL-C, LDL-C, TC/HDL-C: no effect
Navarrete-Cortes et al. (2014) [90]	Mexico	360 mg/day Mg lactate	3 months	T2DM + normomagnesemia	98	TC, HDL-C, LDL-C, TG: no effect
Guerrero-Romero et al. (2015) [91]	Mexico	30 mL/day MgCl_2_ 5% solution ~382 mg of Mg	4 months	Prediabetes + hypomagnesemia	116	HDL-C: ↑ TG: ↓
Solati et al. (2014) [92]	Iran	300 mg/day MgSO_4_	3 months	T2DM	54	LDL-C, non-HDL-C: ↓ TC, HDL-C, TG: no effect
De Valk et al. (1998) [93]	The Netherlands	15 mmol/day Mg aspartate	3 months	Controlled T2DM	50	TC, HDL-C, TG: no effect
Talari et al. (2019) [95]	Iran	250 mg/day MgO	24 weeks	T2DM + HD	54	TC, LDL-C ↓
Afzali et al. (2019) [96]	Iran	250 mg/day MgO + 400 IU/day vitamin E	12 weeks	T2DM + diabetic foot	57	HDL-C: ↑ LDL-C, TG: ↓
Karamali M. et al. (2018) [98]	Iran	200 mg/day Mg + 800 mg/day Ca + 8 mg/day Zn + 400 IU vitamin D	6 weeks	Gestational diabetes	60	VLDL, TG: ↓
Sadeghian et al. (2020) [99]	Iran	250 mg/day MgO	12 weeks	T2DM nephropathy	80	TC, LDL-C, HDL-C, TC/HDL-C, TG: no effect
Farshidi et al. (2020) [105]	Iran	300 mg/day MgSO_4_	6 months	CHD	64	HDL-C: ↑ oxLDL, TC/HDL-C, LDL-C: ↓
Zemel et al. (1990) [109]	USA	40 mmol/day Mg aspartate	3 months	Hypertension	13	TC, HDL-C, LDL-C, TG: no effect
Guerrero-Romero and Rodriguez-Moran (2008) [111]	Mexico	2.5 g/day MgCl_2_ (450 mg Mg)	4 months	Hypertension	82	HDL-C: ↑ TG: no effect
Cunha et al. (2016) [113]	Brazil	1200 mg/day Mg chelate	6 months	Hypertension in women prescribed thiazides	35	HDL-C, LDL-C: no effect
Mortazavi et al. (2013) [123]	Iran	440 mg MgO x3/week	6 months	HD	54	LDL-C: ↓ TC, HDL-C, TG: no effect

Mg, magnesium. RCTs, randomized clinical trials. ↑, increased. ↓, decreased. mg, milligrams. g, grams. USA, United States of America. mL, milliliter. Mg(OH)_2_, Mg hydroxide. MgO, Mg oxide. MgCl_2_, Mg chloride. Ca, calcium. Zn, Zinc. Cr, chromium. IU, international units. MONW, metabolically obese normal-weight. PCOS, polycystic ovary syndrome. NAFLD, nonalcoholic fatty liver disease. MetS, metabolic syndrome. T2DM, type 2 diabetes mellitus. CHD, coronary heart disease. HD, hemodialysis. HDL-C, high-density lipoprotein cholesterol. LDL-C, low-density lipoprotein cholesterol. TC, total cholesterol. TG, triglycerides. VLDL, very low-density lipoprotein cholesterol. apoA1, apolipoprotein A1. oxLDL, oxidized LDL.
